# The HL-60 clone 15 cell line as a model for leukocyte migration–possibilities and limitations

**DOI:** 10.3389/fimmu.2025.1515993

**Published:** 2025-05-27

**Authors:** Lena Griesbaum, Christopher Weusthof, Thorben Sauer, Timo Gemoll, Gregor Weirich, Ludger Klimek, Julie Petry, Ali Bashiri Dezfouli, Maria Shoykhet, Barbara Wollenberg

**Affiliations:** ^1^ Department of Otolaryngology, Head and Neck Surgery, Technical University of Munich, School of Medicine and Health, TUM University Hospital, Munich, Germany; ^2^ Section for Translational Surgical Oncology & Biobanking, Department of Surgery, University Hospital Schleswig- Holstein and University of Lübeck, Lübeck, Germany; ^3^ Institute of Pathology, Section for Cytology, Technical University of Munich, School of Medicine and Health, Munich, Germany; ^4^ Department of Otolaryngology, Head and Neck Surgery, University Medical Centre Mainz, Mainz, Germany; ^5^ Center for Rhinology and Allergology, Wiesbaden, Germany; ^6^ Department of Radiation Oncology, Technical University of Munich, School of Medicine and Health, Central Institute for Translational Cancer Research, Technical University of Munich (TranslaTUM), TUM University Hospital, Munich, Germany

**Keywords:** eosinophilic differentiation, eosinophil biology, eosinophilic research, immunology, allergy, migration, chemotaxis, proteomics

## Abstract

**Introduction:**

As a part of the innate immune system, eosinophils are recruited during infectious diseases, to release their characteristic cytotoxic granules and catch pathogens in extracellular traps. Moreover, eosinophils have a crucial role in autoimmune diseases, for example allergies. The isolation of these densest and lowest abundant leukocytes is cost-and labor intense. This sets restrictions on many aspects of eosinophilic research. In this study, we performed a thorough characterization and functional assessment of the HL-60 clone 15 (HC15) cell line, which can be differentiated into eosinophil-like cells, to investigate its potential in eosinophil research.

**Methods:**

HC15 cells were differentiated with sodium butyrate with or without IL-5 and cells were characterized and compared to primary eosinophils, neutrophils and peripheral blood mononuclear cells. Cell features were analyzed using proteomics, morphologic assessment, RT-qPCR, immunofluorescent staining and flow cytometry. Based on these results, functional tests were performed, including transwell migration assays, flow cytometry-based aggregate formation assays, immunofluorescent microscopy-based adherence assays to endothelial cells and flow cytometry- and ELISA-based activation assays.

**Results:**

The proteomes of the cell line cells differed from those of primary eosinophils and neutrophils. Differentiation of HC15 cells enhanced the expression of GATA-1 and altered the expression of surface markers IL-5R, EMR1, and TREM-1. Differentiated HC15 cells overexpressed the granule protein EPX compared to primary eosinophils and induced a distinct inflammatory milieu by secreting CCL-5, EPX and IL-8. The addition of IL-5 during differentiation increased this effect. Cell line cells responded weaker to activation than primary eosinophils but showed a similar migration and adherence pattern in multiple assays. These features were mostly unaffected by differentiation.

**Discussion:**

Differentiation of HC15 cells induces an eosinophil lineage-committed precursor state. Hence, the differentiated cell line cells lacked characteristic features of eosinophils such as morphologic attributes, surface marker expression and the capacity to be activated. However, the cells were able to migrate, form aggregates with platelets and similarly adhere to endothelial cells as primary eosinophils. It is, therefore, advisable to use the cell line as an eosinophilic model only in research questions related to chemotaxis and migration.

## 1 Introduction

Eosinophils are fully matured myeloid cells with a size of 12-17 µm. They are primarily found in tissues, where they can survive up to 12 days without activation. In the blood, eosinophils make up 1-3% of all peripheral leukocytes (1).

Eosinophils originate from the bone marrow where they develop in a two-step, IL-5-dependent process: lineage commitment and maturation. In the lineage commitment step, a subset of common myeloid precursor cells gains the expression of IL-5 receptor chain α (IL-5Rα), priming them for eosinophilic differentiation (2). This process is regulated by a fine interplay between GATA-binding (GATA) and CCAAT/enhancer-binding protein transcription factors (TFs) (3). Additionally, PU.1, the TF regulating granule protein synthesis is expressed early during eosinophil differentiation (4). Compared to lineage commitment, about twice as many transcriptome alterations occur during the eosinophil maturation step. A large array of TFs orchestrates and tightly regulates this complex process (5). One prominent TF involved is inhibitor of DNA binding 2 (ID2), which supports end stage maturation (6).

Fully matured eosinophils do not proliferate anymore and show characteristic morphologic features, such as bilobed nuclei and a high amount of densely packed granules (3). Moreover, they possess an array of specific surface receptors. In a type 2 inflammatory context C-C chemokine receptor 3 (CCR3) signaling induces eosinophil transmigration (7). IL-5R signaling on the other hand rather enhances eosinophil survival and effector functions (8, 9). Other surface receptors, which are commonly used to identify eosinophils are sialic acid binding Ig like lectin 8 (Siglec-8) and EGF-like module-containing mucin-like hormone receptor-like 1 (EMR1) (10, 11).

Already in the 1980s, eosinophils were recognized to mainly assist host-defense against bacterial, fungal or viral threats (12, 13). In accordance, inflammatory diseases, such as helminth infection, allergic diseases and some autoimmune disorders are accompanied by tissue and/or blood eosinophilia (14–18). As cytotoxic effector cells of the innate immune system, eosinophils are rapidly recruited to sites of inflammation, infection or allergen exposure where they release a variety of molecules, such as cytokines, chemokines, growth factors and cationic granule proteins (19). Additionally, eosinophils are able to release DNA to form extracellular traps which can fix pathogens for later clearance (20, 21).

Leukocyte recruitment involves a series of different steps. Initially, endothelial cells expose adhesion receptors as a result of activation. Subsequently, circulating leukocytes get captured, firmly adhere, crawl along the endothelium and extravasate with the help of adhesion receptors on both sides (22). An important aspect directing this process is chemotactic stimulation (23). Moreover, platelets (PLTs) were recently found to assist leukocyte recruitment. Activated PLTs binding to the endothelium provide adherence receptors for leukocyte tethering and activation, guiding them to their extravasation site (24, 25).

Eosinophils are the densest and lowest abundant leukocytes. For their isolation, a discontinuous multiple density Percoll gradient centrifugation protocol was developed yielding 38-56% recovery (26). To increase the purity of the isolated cells, this method is often coupled with negative selection by magnetic separation, adding cost and labor but not increasing the recovery rate (27). Additionally, a high prevalence of internal RNAses and fast degranulation even after mild triggers set limits to the usability of primary eosinophils *in vitro* (28).

A widely used cell line in eosinophil research is HL-60 clone 15 (HC15), a variant of the human promyelocytic leukemia cell line HL-60, first published in the 1980s. By culture continued culture in slightly alkaline conditions (pH 7.6-7.8) for 2 months the cells gained the potential differentiate into eosinophils if stimulated with 0.5 mM sodium butyrate (SB) for 5–7 days (29, 30). The histone deacetylase inhibitor butyrate induces the continuous acetylation of histones H3 and H4, resulting in eosinophilic features, such as the expression of CCAAT/enhancer-binding protein TFs, eosinophil major basic protein (EMBP) and the expression of β7 integrin (31).

However, the consolidation of the HC15 cell line as a model for functional immune cells is still weak. Their similarity to primary eosinophils is still unknown in many aspects. Hence, most studies using HC15 cells as eosinophilic cells only investigated functions such as chemotaxis (32–34) or granule protein expression (28, 35, 36) in a very isolated manner. Moreover, a heterogenous array of methods evaluating differentiation efficacy were used in the past. While some studies did not evaluate differentiation (28, 32–35, 37), other studies investigated morphologic changes with different histologic staining methods (29–31, 38), and one study used other criteria, such as granule protein expression to determine eosinophil-likeness (39).

This study aims to characterize the HC15 cell line in detail and to find suitable markers for eosinophilic cell differentiation. To increase the value of the HC15 cell line for eosinophil research, but also to demonstrate limitations, we implemented a wide array of methods such as proteomics, flow cytometry, histologic and immunofluorescent staining, as well as RT-qPCR to measure eosinophil-specific characteristics in the HC15 cell line and compared it to human primary eosinophils (Eos). Furthermore, we studied the role of IL-5, which has been added during eosinophil differentiation by some groups, which has not yet been studied in detail (32, 35, 40).

## 2 Materials and Methods

### 2.1 HC15 cell culture and differentiation

All cells were cultured at 37°C and 5% CO_2_. HC15 cells (ATCC-CRL-1964) were purchased from LGC Standards GmbH (Teddington, U.K.). Cells were cultured at 0.25 to 1.5 x 10^6^ cells/mL in RPMI 1640 (72400054, Thermo Fischer Scientific Inc., Waltham, MA, USA) (10% FBS (F7524, Merck KGaA, Darmstadt, Germany), 1% P/S (P4333, Merck), pH 7.6-7.8) with half medium changes done every day to ensure slightly alkaline conditions. For differentiation, a full medium change was performed. Cells were centrifuged for 5 min at 300 g, washed once with PBS and resuspended at 5 x 10^5^ cells/mL in RPMI 1640 (10% FBS, 1% P/S, pH 7.6-7.8) supplemented either with 0.5 mM SB (sc-202341, Santa Cruz Biotechnology, Inc., Dallas, TX, USA) (DHC15) or with 0.5 mM SB and 10 ng/mL IL-5 (NBP2-34897, Biotechne GmbH, Wiesbaden Nordenstadt, Germany) (IHC15) for 5 days, in line with what has been described before (32, 35, 40). For proteomics measurements, 50 ng/mL IL-5 were used to differentiate IHC15 cells. During differentiation, no medium change was performed. If not indicated differently, cells were incubated at 1 x 10^6^ cells/mL for all experiments. Activation was performed in fresh medium with 10 µM phorbol-12-myristate-13-acetate (PMA, P1585, Merck) for 90 min.

### 2.2 Isolation of Eos

Venous blood from self-proclaimed healthy volunteers was collected into tubes containing 3.2% sodium citrate (SAR-011606001, Sarstedt, AG & Co. KG).

Eos were isolated from 50 mL whole blood from healthy volunteers using a customized protocol. In brief, erythrocytes were sedimented in 10 mL batches using the Sedimentation Kit II (130-126-357, Miltenyi Biotech B.V. & Co. KG, Bergisch Gladbach, Germany). Each resulting pellet was resuspended in 200 µL MACS^®^ Separation Buffer (130-091-221, Miltenyi Biotech), with 80 µL Erythrocyte Depletion Microbeads from the human StraightFrom^®^ Whole Blood peripheral blood mononuclear cell (PBMC) Isolation Kit (130-126-359, Miltenyi Biotech) and 40 µL human CD61 Microbeads (130-051-101, Miltenyi Biotech) and incubated for 10 min at 4°C for magnetic labelling. Magnetic separation was performed as described in the human StraightFrom^®^ Whole Blood PBMC Isolation Kit. Eosinophils were isolated from the pooled flow throughs using the human Eosinophil Isolation Kit (130-092-010, Miltenyi Biotech). Quality control was done using flow cytometry by staining with CD45-APC/Cyanine7 (2D1, mouse IgG1, 368516, Biolegend Inc., San Diego, CA, USA), Siglec-8-PE (mouse 7C9, IgG1, 347104, Biolegend) and CD16-BV 510™ (B73.1, mouse IgG1, 360730, Biolegend) (for gating see **Supplementary Figure 1A**). This protocol resulted in a recovery of up to 2.14 x 10^5^ cells/mL of whole blood (median 5.46 × 10^4^ cells/mL, 95% confidence limits: [5.03 x 10^4^ cells/mL – 7.87 x 10^4^ cells/mL], data not shown) and a median eosinophil (= CD45^+^Siglec-8^+^) purity of 97.3% [94.8% - 97.2%] (**Supplementary Figure 1B**). For proteomics, Eos were isolated using the MACSxpress Eosinophil isolation kit (130-104-446, Miltenyi Biotech) followed by a magnetic erythrocyte depletion kit (130-098-196, Miltenyi Biotech), which resulted in a recovery of up to 2.07 x 10^4^ cells/mL whole blood (median 9.40 x 10^3^ cells/mL [8.93 x 10^2^ cells/mL – 2.22 x 10^4^ cells/mL]) and a median purity of 99.2% [98.5% - 99.9%] (data not shown). If not indicated differently, cells were incubated at 1 x 10^6^ cells/mL for all experiments. Activation was performed in fresh medium with 10 µM PMA for 90 min.

### 2.3 Isolation of human primary neutrophils (Neutros)

Neutros were isolated from 8 mL whole blood from healthy volunteers using the MACSxpress^®^ whole blood neutrophil isolation kit (130-104-434, Miltenyi Biotech) followed by magnetic erythrocyte depletion. The manufacturers’ instructions were followed and quality control was done using flow cytometry by staining with CD45-APC/Cyanine7, Siglec-8-PE and CD16-BV 510™. Median neutrophil (= CD45^+^CD16^+^) purity was 99.6% [99.4% – 99.9%] over all experiments (data not shown).

### 2.4 Isolation of PBMCs

PBMCs were isolated from 50 mL whole blood from healthy volunteers. In brief, blood was layered over Ficoll-Paque™ Plus (17144003, Cytiva, Marlborough, MA, USA) in a 2:1 ratio and centrifuged at 800 g for 30 min without brake. Subsequently, the enriched PBMC layer was collected. Isolated PBMCs were washed with PBS prior to further use.

### 2.5 Proteomics

#### 2.5.1 Sample acquisition

Protein extraction for proteomics was performed using the EasyPep™ Mini MS Sample Prep Kit (A40006, Thermo Fischer Scientific) following the manufacturers’ instructions. In the case of cell line cells, 1 x 10^6^ cells were lysed with 100 µL lysis buffer. In the case of Eos or Neutros, all cells gained from 50 mL and 10 mL of whole blood were used. Due to low Eos yields from peripheral blood, primary cells from three donors were pooled in 100 µL lysis buffer, respectively. Protein concentrations were measured using the Roti^®^ Nanoquant Bradford solution (K880.1, Carl Roth). Four samples of cell line cells were processed as separate replicates, whereas two pooled Eos or Neutros samples were processed as three technical replicates, respectively, to account for pooling.

#### 2.5.2 Liquid chromatography coupled mass spectrometry

The samples were solubilized with a final concentration of 1 µg/µL in solvent A (0.1% formic acid) and were loaded into a HPLC Dionex Ultimate 3000 (Thermo Fischer Scientific). The samples were first loaded onto a trap column (μ-Precolumn Acclaim PepMap100, internal diameter: 0.3 x 5 mm, 5 μm, 100 Å, Thermo Fischer Scientific) and desalted with loading solution at 10 μL/min for 4 min. Peptides were subsequently separated using an analytical column (LC Column, 3 μm C18 (2), 0.3 x 50 mm, 3 μm, 100 Å, Phenomenex Inc., Torrence, CA, USA) and eluted with a multi-step gradient of solvent B (0.1% formic acid in acetonitrile) in solvent A for 86 min at a flow rate of 5 µL/min. Purified peptides were analyzed with a TripleTOF 5600+ mass spectrometer (AB ScieX, Framingham, MA, USA). The following SWATH acquisition working parameters were used: Ion Spray Voltage Floating at 5000 V; ion source gas, 15; ion source gas, 0; curtain gas at 30 and source temperature heating set to 0°C. The optimized declustering potential was set at 100; collision energy to 19.2; collision energy spread, 5.0; ion release delay, 67; ion release width at 25. For data acquisition, one 0.049965 s MS scan (m/z 350–1250) was performed, followed by 100 variable Q1 windows with the size range 5–91.3 Da, each at 0.030 s accumulation time with CES at 5 eV. The precursor isolation windows were defined using the SWATH Variable Window Calculator V1.1 (AB Sciex) based on precursor *m*/*z* densities obtained from DDA spectra. For DDA acquisition, identical instrument working parameters were used. MS scans were performed for 350–1250 Da with an accumulation time of 0.25 s, MS/MS scans were performed for 100–1500 Da with an accumulation time of 0.05 s at high sensitivity mode.

#### 2.5.3 SWATH data processing

The raw SWATH data were processed using the software tool DIA-NN v1.8.1 developed by Vadim Demichev et al. (41). The software was used in the high-accuracy LC mode with RT-dependent cross-normalization enabled. Mass accuracy, MS1 accuracy, and scan window settings were set to 0, as DIA-NN optimizes these parameters automatically. The ‘match between runs’ function was used first to develop a spectral library using the ‘smart profiling strategy’ from the data-independent acquisition data. The human UniProtKB/swiss-prot database (version 2020/12/6) (42) was used for protein inference from identified peptides. Trypsin/P was specified as protease. The precursor ion generation settings were set to peptide length of 7–52 amino acids, the maximum number of missed cleavages to one. The maximum number of variable modifications was set to 0. N-terminal methionine excision and cysteine carbamidomethylation were enabled as fixed modifications. The protein group matrix output containing normalized MaxLFQ (43) quantities was used for further analysis.

#### 2.5.4 Proteomics data analysis

To assess the equivalence between the cell line cells and Eos, a two one-sided t-test (TOST) between the IHC15 and Eos proteomes was performed using the tool Jamovi version 2.3.18 with the equivalence testing package “TOSTER”. P-values were calculated by Welch`s t-test with a significance level of p < 0.05. The overall clustering of all samples was visualized using a principal component analysis (PCA), conducted using the tool Perseus version 4.1.3.0.

Differential protein abundance testing was conducted in R version 4.0.3 – 4.3.3. with the packages “limma, “dplyr” and “readr”, using Bayes moderation and Benjamini-Hochberg correction to calculate false discovery rates. Differentially abundant proteins (DAPs) between Eos and IHC15 cells as well as Neutros and IHC15 cells were visualized in volcano blots using the package “EnhancedVolcano”. DAPs were considered significant with an FDR < 0.05 and a |log_2_-fold change| > 1. Using the differential protein abundance data from IHC15 cells and Eos, a gene set variation analysis (GSVA) was performed using the package “gsva”. Shortly, this analysis uses log_2_-fold changes and p-values to rank proteins according to their impact on overall proteome differences. From all proteins in a specific gene set, a score is computed indicating the overall impact of this gene set on overall differences between two proteomes. The used gene sets (BIOCARTA_EOSINOPHILS_PATHWAY, GOBP_EOSINOPHIL_DIFFERENTIATION, GOBP_EOSINOPHIL_CHEMOTAXIS, GOBP_EOSINOPHIL_MIGRATION, GOBP_EOSINOPHIL_ ACTIVATION, GOBP_EOSINOPHIL_MEDIATED_IMMUNITY) were chosen to encompass a broad spectrum of eosinophilic functions and were assessed on the Molecular Signatures Database (downloaded November 2022). The GSVA results were visualized using the package “ggplot2”.

Utilizing the package “Venn Diagram” in R and lists of gene IDs of all proteins found within one condition, Venn diagrams were plotted overlapping proteins between samples. From these Venn diagrams, gene IDs of proteins exclusively shared between IHC15 and Eos, between DHC15 and Eos or only abundant in Eos were extracted and analyzed in a pathway enrichment analysis utilizing the tool Metascape version 3.5 (https://metascape.org) (44). This tool uses lists of geneIDs to carry out a functional enrichment analysis. In brief, pathways are identified as enriched if the number of found associated genes exceeds the number which is expected by chance. This is evaluated using a hypergeometric distribution test followed by Benjamini-Hochberg correction. The resulting pathways are clustered based on similarity and the pathway with the lowest p-value from each cluster is represented in a bar graph. Lists of the respective geneIDs used for pathway analysis can be found in the **Supplementary Material** (**Supplementary Tables 1**-**3**).

### 2.6 Reverse transcription quantitative polymerase chain reaction (RT-qPCR)

RNA was isolated using the *mir*Vana miRNA Isolation Kit (AM1561, Thermo Fischer Scientific) following the manufacturers’ protocol. For lysis of 1 x 10^6^ cells, 600 µL Lysis solution and 60 µL mRNA homogenate additive were used. RNA extraction was done using 600 µL Acid-Phenol: Chloroform, pH 4.5 (with IAA, 125:24:1, AM9722, Thermo Fischer Scientific). RNA was eluted with 100 µL hot nuclease free water (95°C) and the final RNA concentration was measured on a Nanodrop 2000 (Thermo Fischer Scientific). Reverse transcription into cDNA was performed using the LunaScript^®^ RT SuperMix (E3010L, New England Biolabs GmbH, Ipswich, MA, USA) on a DNA-engine Thermal cycler (Bio-Rad Laboratories GmbH, Hercules, CA, USA). In a 20 µL reaction, 250 ng RNA were transcribed resulting in a final concentration of 12.5 ng/µL cDNA. For running the qPCR, the Luna^®^ Universal qPCR Master Mix (M3003L, New England Biolabs) was used. In each reaction, 12.5 ng cDNA template (1 µL) were employed. The primers used for target gene recognition are depicted in **Table 1**. The RT-qPCR was run on a Lightcycler^®^ 96 (F. Hoffmann-La Roche Ltd, Basel, Switzerland) and the RNA expression level for each target gene was calculated as 2^-ΔΔCT^.

**Table 1:** Targets and primers used for RT-qPCR analyses.

Gene symbol	Protein encoded by gene	Accession number	Forward and reverse primer	Product size (bp)	Exon spanning
***ADGRE1***	EGF-like module-containing mucin-like hormone receptor-like 1 (EMR1)	NM_001974.5	5’-CACCTGTGAAGACGTGGAT-3’ 5’-ACACGATGCTTTGAGACCCT-3’	154	YES
***B2M***	Beta-2-microglobulin	NM_004048.4	5’-ATGAGTATGCCTGCCGTGTG-3’ 5’-TCTGCTCCCCACCTCTAAGT-3’	326	YES
***CD40***	CD40 Molecule	NM_001250.6	5’-TGATGTTGTCTGTGGTCCCC-3’ 5’-GCTTCTTGGCCACCTTTTTGA-3’	119	YES
***CD63***	CD63 Molecule	NM_001780.6	5’-TTGCTTTTGTCGAGGTTTTGGG-3’ 5’-CCAGAGGACAGGGAACATCAG-3’	243	YES
***EPX***	Eosinophil peroxidase	NM_000502.6	5’-CCTACCGAGACTTTCTGCCC-3’ 5’-GGTCGATGCCCCCTTCATAC-3’	261	YES
***GATA1***	GATA binding protein 1	NM_002049.4	5’-CCAAGAAGCGCCTGATTGTC-3’ 5’-CATCCTTCCGCATGGTCAGT-3’	171	YES
***ID2***	Inhibitor of DNA binding 2	NM_002166.5	5’-CCTGTCCTTGCAGGCTTCTGA-3’ 5’-ACAGTCCAAGTAAGAGAACACCC-3’	269	YES
***IL5RA***	Interleukin 5 receptor subunit alpha	NM_175726.4	5’-CTGTGCCTGACGCTATGCTA-3’ 5’-ACAGGTGGGAGAAGTGAAATCTT-3’	291	YES
***ITGAL***	Integrin subunit alpha L (CD11a)	NM_002209.3	5’-ACTTTGGATACCGCGTCCTG-3’ 5’-CAGGGTCACAGGCCAAAATG-3’	219	YES
***ITGAM***	Integrin subunit alpha M (CD11b9	NM_000632.4	5’-GCAGCATCAATATCAGGTCAGC-3’ 5’-CAGCGATGGAGCAGTTCACC-3’	224	YES
***PRG2***	Eosinophil major basic protein (EMBP)	NM_002728.6	5’-GGTCTCTGGGTGGGATAAAG-3’ 5’-AGGGGTCTCAAAGGTGGAAG-3’	112	YES
***SELPLG***	Selectin P ligand (PSGL-1)	NM_003006.4	5’-CATTGGGGGTTGCTCGGAT-3’ 5’-CAGAGGCATGGCACCACC-3’	160	YES
***SIGLEC8***	Sialic acid binding Ig like lectin 8	NM_014442.3	5’-AGACGCCAGGAAGAGGGATA-3’ 5’-CTATGGGTCAGGGCTGTCA-3’	132	YES
***SLC44A2***	Solute carrier family 44 member 2	NM_020428.4	5’-CTACGGGAAACACGGAACGC-3’ 5’-CACCTTTCGAGGGTCTCCAT-3’	166	YES
***SPI1***	Spi-1 proto-oncogene (PU.1)	NM_003120.3	5’-AGATGCACGTCCTCGATACC-3’ 5’-CTTCTTCTTGCTGCCTGTCTC-3’	233	YES
***TREM1***	Triggering receptor expressed on myeloid cells 1	NM_018643.5	5’-TCCTCCTACCACCACTAAGG-3’ 5’-GAACACCGGAACCCTGATG-3’	151	YES

### 2.7 Hemacolor^®^ staining

For leukocyte staining, the cells obtained from the human StraightFrom^®^ Whole Blood PBMC Isolation Kit before isolating eosinophil with the human Eosinophil Isolation Kit were used. After washing twice with PBS, 1 x 10^6^ cells were cytospun on a glass slide for 5 min at 200 g, fixed with M-Fix^®^ fixation spray (1039810102, Merck) and airdried at 37°C for 5 min. Cells were stained using the Hemacolor^®^ staining kit (111661, Merck) according to the manufacturers’ instructions and mounted using Eukitt mounting medium (03989, Merck) Microscopy was performed on an Axioscope connected to an AxioCam ERc5s (Carl Zeiss Meditec AG, Oberkochen, Germany) with a magnification of 40 x. On each slide, 5 pictures of randomly chosen areas were taken.

### 2.8 Flow cytometry

Prior to antibody staining, cells were fixed with 0.5% paraformaldehyde (PFA, P6148, Merck) in PBS for 15 min at room temperature. Subsequently, cells were washed with PBS by centrifugation for 5 min at 300 g and resuspended at 1 x 10^6^ cells/mL in FACS buffer (1% FBS, 2 mM EDTA (V4231, Promega GmbH, Walldorf, Germany) in PBS). Unspecific binding was blocked by incubation with TruStain FcX™ (422301, Biolegend) for 10 min at room temperature. Cells were washed and incubated with monoclonal antibodies α-CD193 (CCR3)-PE (5E8, mouse IgG2b, 310705, Biolegend), α-CD11b- BV 510™ (ICRF44, mouse IgG1, 301333, Biolegend), α-CD63-PB™ (H5C6, mouse IgG1, 353012, Biolegend), α-CD41-PE (HIP8, mouse IgG1, 303705, Biolegend) and α-CD62P-BV 510 (AK4, mouse IgG1, 304936, Biolegend) or isotype controls PE mouse IgG2b (MPC-11, 400314, Biolegend), BV 510™ mouse IgG1 (MOPC-21, 400172, Biolegend), PB™ mouse IgG1 (MOPC-21, 400151, Biolegend) for 15 min at room temperature in the dark. After washing, cells were measured using a CytoFLEX flow cytometer (Beckman Coulter GmbH, Krefeld, Germany). Data analysis was performed using Flow Jo v10.10.0.

### 2.9 Immunofluorescence staining and microscopy

For immunofluorescence staining, 12-well chamber slides (81201, Ibidi GmbH, Gräfelfing, Germany) were precoated with Cell-Tak (354240, Corning Inc., Corning, NY, USA) as indicated in the manufacturers’ protocol. Subsequently, 2 x 10^5^ cells in 150 µL RPMI 1640 were added to each chamber and incubated at 37°C for 30 min. Between each of the following steps, cells were washed 3 x 3 min in PBS. The attached cells were fixed using 4% PFA in PBS, permeabilized with 0.2% Tween^®^ 20 (#9127.2, Carl Roth) in PBS and blocked using 2% bovine serum albumin (BSA) (8076.4, Carl Roth), 2% goat serum (ab-7481, Abcam plc., Cambridge, Great Britain), 0.01% Tween^®^ 20 in PBS. All antibodies and dyes were diluted in antibody diluent (0.1% BSA, 0.01% Tween^®^ 20 in PBS). Incubation with the primary antibodies α-EMBP (1:100, rabbit, PA5-102628, Thermo Fischer Scientific) and α-eosinophil peroxidase (EPX) (1:200, AHE-1, mouse IgG1, MAB1087, Merck) was performed overnight at 4°C, followed by incubation with the corresponding secondary antibodies (α-mouse AF488 (1:2 000, goat, A-11001, Thermo Fischer Scientific) and α-rabbit APC (1:250, goat, A10931, Thermo Fischer Scientific)) for 1 h at room temperature in the dark. Next, cells were stained with 2.5 µg/mL wheat germ agglutinin CF^®^ 568 (29077-1, Biotium, San Francisco, CA, USA) and 125 ng/mL DAPI (6335.1, Carl Roth). The slides were airdried, the silicone chambers were removed and the slides were mounted using Prolong™ Gold Antifade Mountant (P10144, Thermo Fischer Scientific). Stained slides were stored at 4°C until analysis on a Thunder Imager (Leica Microsystems, Wetzlar, Germany) with a magnification of 40 x. Of each chamber, 5 pictures of randomly chosen areas were taken.

### 2.10 Transwell migration assay

Cell migration capacity was assessed using a 24-well transwell system (6.5 mm Transwell^®^ with 3.0 μm PC membrane insert, CLS-48EA, Corning). To the upper chamber, 3 x 10^5^ cells in 100 µL RPMI 1640 (10% FBS, 1% P/S, pH 7.6-7.8) were added. If indicated, adherence receptors on eosinophilic cells were blocked in the upper chamber as follows: CD63 was blocked using 5 µg/mL α-CD63-PE (H5C6, mouse IgG1, 353003, Biolegend). CD11a was blocked using 5 µg/mL α-CD11a (HI111, mouse IgG1, 301202, Biolegend). CD11b was blocked using 5 µg/mL α-CD11b (ICRF44, mouse IgG1, 301302, Biolegend). PSGL-1 was blocked using 50 µg/mL α-CD162 (KPL1, mouse IgG1, 328802, Biolegend). SLC44A2 was blocked using 20 µg/mL α-SLC44A2 (rabbit, LS−C750149, Vector Laboratories, Inc., Newark, CA, USA). TREM-1 was blocked using 10 µM LP17 (MedChemExpress, Monmouth Junction, NJ, USA). LP17 was incubated on cells for 2 h at 37°C and in the last 10 min of incubation, all blocking antibodies were added to their respective samples. The indicated antibody concentrations were determined by titration and the lowest blocking concentration was used. Subsequently, 100 nM N-formyl-Met-Leu-Phe (fMLP, F3506, Merck) was supplied to the lower chamber of the transwells. The cells were incubated with fMLP for 1 h at 37°C. In order to detach the migrated cells from the lower face of the membrane, 3 µM EDTA was added to the lower chamber and the cells were incubated for an additional 5 min at 37°C, before the transwells were removed. The remaining medium in the lower chambers was mixed thoroughly and the migrated cells were counted in a Neubauer improved counting chamber in triplicate for each sample or by counting events/µL in 200 µL medium using a flow cytometer. The relative number of migrated cells was determined as a ratio of migrated/total cells.

### 2.11 Isolation of PLTs

PLTs were isolated from 50 mL whole blood from healthy volunteers as described before (45). In brief, blood was centrifuged to collect platelet-rich plasma. After addition of prostaglandin I_2_ and dilution with Tyrode buffer, washed platelets were collected in a second centrifugation step. The platelet pellet was resuspended in Tyrode buffer and quality control and platelet counting were performed using flow cytometry by staining with α-CD45-APC (HI30, mouse IgG1, 304012, Biolegend), α-CD41-AF488 (HIP8, mouse IgG1, 303724, Biolegend) and α-CD62P-BV 510. The median PLT (= CD41^+^) purity was 99.5% [99.2% - 99.7%] with a mean baseline activation (= CD41^+^CD62P^+^) of 0.570% [0.410% - 0.740%] (Data not shown).

### 2.12 PLT-cell line aggregate formation

PLTs were activated by incubation with 25 ng/mL thrombin receptor activator peptide 6 (TRAP, HY-P0078, MedChemExpress) at 1 x 109 cells/mL for 5 min. Cells were resuspended to 1 x 10^6^ cells/mL in 100 µL fresh medium. If indicated, adherence receptors on eosinophilic cells were blocked prior to the addition of platelets as described the transwell migration assay section. Subsequently, 2 x 10^7^ resting or activated PLTs were added without washing for a cell to PLT ratio of 1:200. After 15 min of incubation cells were stained as described in the flow cytometry section. PLT-eosinophilic cell-aggregates were identified as CD41/CD45-double positive events.

### 2.13 Cell adherence to human umbilical vein endothelial cells (HUVECs) in a PLT-rich environment

24-well plates were coated with 0.2% porcine gelatin (G6144, Merck) in Hank’s balanced salt solution (14175095, HBSS; Thermo Fischer Scientific). The gelatin was aspirated and the plates were airdried prior to addition of cells. HUVECs were cultured in Endothelial Cell Growth Medium 2 (C-22011, PromoCell GmbH, Heidelberg, Germany). For experiments, HUVECs were seeded at 1 x 10^5^ cells/well in EM2 into coated 24-well plates. In case of stimulation, 100 pM IL-4 (200-04, Thermo Fischer Scientific) and 100 pM TNFα (11343015, ImmunoTools GmbH, Friesoythe, Germany) were added during seeding. The cells were grown to confluency for 24 h at 37°C, 5% CO_2_. Eos and cell line cells were prestained with 2.5 µM carboxyfluoresceinsuccinimidylester (CFSE, C34570, Thermo Fischer Scientific) in HBSS for 5 min at room temperature in the dark. After staining, cells were washed twice with HBSS, resuspended at 1 x 10^6^ cells/mL in HBSS and added to washed HUVECs at 500 µL per well. If indicated, 5 x 10^7^ PLTs were added to respective wells for a cell to PLT ratio of 1:100 and cells were incubated for 30 min at 37°C. After adherence, the supernatant was carefully removed and wells were washed twice with HBSS. Cells were fixed, permeabilized and stained with DAPI as described in the microscopy section. For microscopy, wells were filled with 500 µL PBS. Stained plates were stored at 4°C. Microscopy was performed on a Thunder Imager (Leica Microsystems, Wetzlar, Germany) with a magnification of 40 x. CFSE^+^ Cells were counted in 10 randomly chosen sections of each well. In some experiments, adherence receptors on eosinophilic cells were blocked prior to their addition to HUVEC cells as described in the transwell migration section. In these cases, the experiment was performed in 96-well plates with 2 x 10^4^ HUVECs/well and 10^5^ eosinophilic cells and the addition of 10^7^ PTLs. For microscopy, wells were filled with 100 µL PBS and cells were counted in 5 randomly chosen sections of each well.

### 2.14 Enzyme-linked immunosorbent assay (ELISA) and Legendplex assay

Cells were centrifuged 300 g for 5 min at room temperature and supernatants from 1 x 10^6^ cells were collected. The following kits were used to determine the secretion levels of a total of 11 biomarkers: Custom Legendplex™ (IL-5 (740043), IL-4 (740540), IL-13 (740047), IL-2 (740934)) (1:1, Biolegend), Human IL-33 DuoSet ELISA (DY3625B, 1:1, Bio-techne), Human CCL11 (C-C chemokine 11/Eotaxin) DuoSet ELISA (DY320, 1: 1, Bio-techne), Human IL-6 DuoSet ELISA (DY206, 1: 1, Bio-techne), Human IL-8 ELISA Max™ Deluxe Set (431504, 1: 1, Biolegend), Human CCL5 (C-C chemokine 5/regulated on activation, normal T-cell expressed and secreted (RANTES)) ELISA Max™ Deluxe Set (440804, 1: 3, Biolegend), Human IL-12 (p70) ELISA Max™ Deluxe Set (431704, 1: 1, Biolegend), Human EPX ELISA Kit (NB-E11396A, 1:50 (cell line), 1:1 (Eos), Novatein Biosciences Inc., Woburn, MA, USA). All experiments were run as described in the manufacturers’ protocols with diluting the samples as indicated.

### 2.15 Statistical analysis

Prior to statistical analysis, outliers were removed in Graphpad prism version 10.2.3 using the ROUT method with the Q value set to 1%.

Subsequently, statistical analyses were computed in R version 4.3.3 using the package “rstatix”. Due to the small sample sizes all data was assumed to be non-normally distributed. All tests were computed in an unpaired manner. Two groups with one variable were compared using a Mann-Whitney U test. For more than two groups with one variable, Kruskal-Wallis testing followed by Dunn’s multiple comparisons with Bonferroni correction was performed. Due to the lack of a fitting non-parametric alternative testing strategy, statistical significance between more than two groups with more than one variable was determined by two-way ANOVA followed by Tukey’s multiple comparisons with Bonferroni correction. In the case of two groups with more than one variable, while all possible multiple comparisons were computed, only comparisons untreated vs. treated within one differentiation protocol (between treatments) or between differentiation protocols of one treatment (between differentiations) were depicted in the graphs. Statistical significance was assumed with a *p*-value ≤0.05.

For all data, effect sizes with 95% confidence intervals were computed in R using the packages “effsize” and “apaTables”. For Mann-Whitney U tests, Cliff’s delta was used, for Kruskal-Wallis testing, Eta^2^ was calculated and for two-way ANOVA, partial Eta^2^ was determined. Details on all significances, multiple comparisons and effect sizes with confidence intervals are depicted in **Supplementary Tables 4**-**11**.

Graphs were plotted using Graphpad prism version 10.2.3. The data is depicted as boxplots with a horizontal line at the median, the 25th to 75th percentiles as hinges and whiskers extending to the lowest and highest datapoints. N depicts the number of independent experiments in each experiment, representing cells of one passage or one healthy donor, respectively.

## 3 Results

### 3.1 The proteome of differentiated HC15 cells is similar to eosinophils, especially in differentiation, chemotaxis and migration pathways

**Figure 1:**
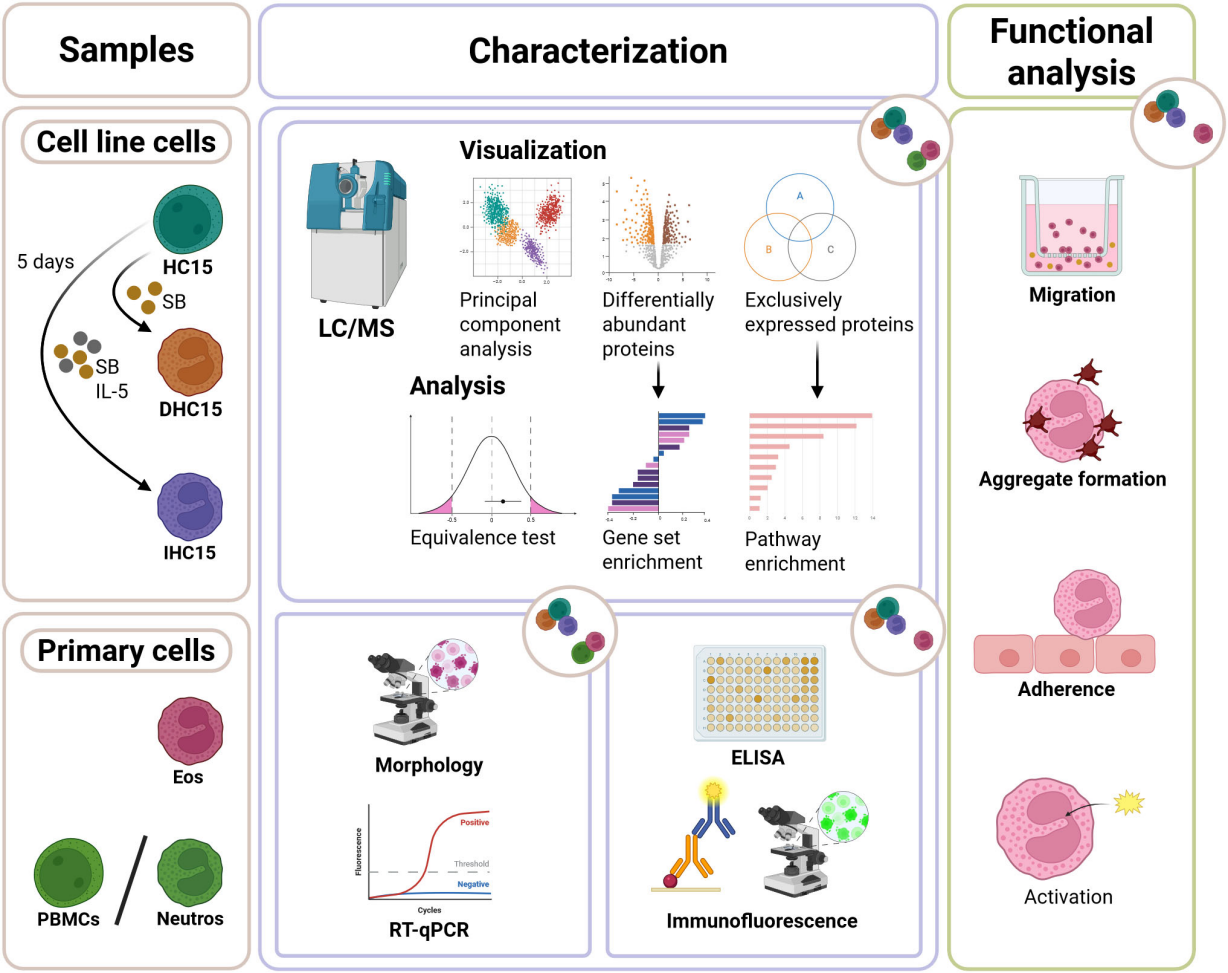
Experimental scheme showing the workflow of studying the eosinophilic cell line HC15. The cell line cells were cultured at pH 7.6-7.8. For differentiation, they were incubated with 0.5 mM SB for 5 days with or without the addition of 10 ng/mL IL-5. The cells were compared to primary Eos and Neutros in a comprehensive proteomics approach. Subsequently, the cell line cells were characterized using different techniques such as RT-qPCR, ELISA and staining methods paired with microscopy. Lastly, their similarity to Eos was tested in functional assays such as migration, aggregation, adherence and activation assays.

To determine the value of HC15 cells as a model for eosinophilic research, in this study we evaluated the two most commonly used differentiation methods, namely SB = DHC15 and SB+IL-5 = IHC15 and compared the cell line to Eos, Neutros and PBMCs in a set of descriptive and functional assays (see **Figure 1** for the workflow of differentiation, as well as for an overview over all conducted tests and analyses). To assess the biological differences of the differentiated cell line and Eos, we performed proteomics, followed by TOST test and found the proteomes of the IHC15 cells and Eos to be significantly similar (**Table 2**).

**Table 2:** TOST comparison of the proteomes from IL-5-differentiated cells vs. Eos.

TOST results			
	t	df	p
t-test	2.57	7019	0.010
TOST Upper	2.59	7019	0.005
TOST Lower	2.56	7019	0.995

Equivalence Bounds		
	Low	High
Hedges**’**s g(av)	-3.31e-4	3.31e-4
Raw	-0.500	0.500

Effect sizes			
		90% Confidence Interval	
	Estimate	Lower	Upper
Hedges**’**s g(av)	0.492	0.0178	0.0806
Raw	74.3	26.8	122

Descriptives					
	N	Mean	Median	SD	SE
Eos	5484	218	15.4	1998	27.0
IHC15	5484	144	24.2	755	10.2

Welch’s t-test.

Denominator set to the average SD.

**Figure 2:**
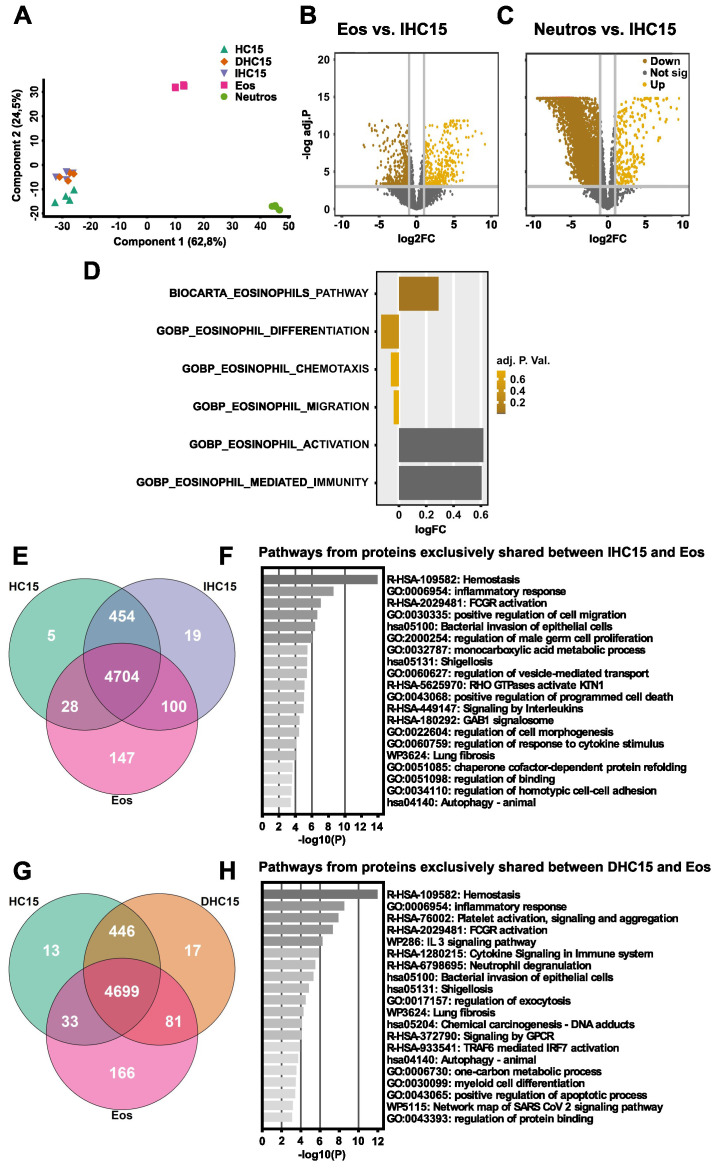
The proteome of IL-5-differentiated cells resembles eosinophils in differentiation, chemotaxis, and migration pathways, but not in immunity and activation pathways. The proteomes of HC15 (N=4), DHC15 (N=4), IHC15 (N=4), Eos (N=6) and Neutros (N=6) were measured using a shotgun proteomics approach. **(A)** PCA comparing all measured groups produced using the Perseus software version 4.1.3.0. **(B, C)** Volcano blot of DAPs in **(B)** Eos vs. IHC15 or **(C)** Neutros vs. IHC15. The data were plotted using R version 4.0.3. DAPs were considered significant with an FDR <0.05 and a log_2_FC of >1. **(D)** GSVA of DAPs in IHC15 vs. Eos. The used gene sets were downloaded from the Molecular signatures Database. **(E, F)** Venn diagrams of gene IDs found in **(E)** HC15 vs. IHC15 vs. Eos or in **(F)** HC15 vs. DHC15 vs. Eos. The graphs were produced using R version 4.3.3. **(G, H)** Pathway enrichment analysis of exclusively shared proteins between **(G)** IHC15 and Eos or **(H)** DHC15 and Eos computed using Metascape (44). The data were analyzed and visualized using R version 4.0.3.-4.3.3.

We clustered the proteomes using a PCA, to analyze the data in more detail (**Figure 2A**). Generally, all cell line samples clustered together independent of their differentiation status, closer to Eos than to Neutros in the first component. A list of the 15 highest contributing proteins in component 1 is given in **Supplementary Table 12**. Out of the 11 highest contributing proteins were 5 eosinophilic or neutrophilic granule proteins, namely lactotransferrin, eosinophil cationic protein, neutrophil defensin 3, azurocidin and neutrophil elastase. In addition, several adhesion proteins highly contributed to the similarities of the cell line cells to Eos. In contrast, in component 2 the cell line cells were more similar to Neutros than to Eos. Nonetheless, differentiation of the HC15 cell line induced a slight shift towards Eos in this component. Out of the top 15 contributing proteins in component 2, many were intracellular signaling molecules contributing to different generic cellular pathways (**Supplementary Table 13**).

The observations made in the PCA analysis were also reflected in a differential protein abundance depicted in volcano blots comparing the proteomes of IHC15 cells and Eos or Neutros, respectively. These showed a higher number of DAPs between the IHC15 and Neutros proteomes than between the IHC15 and Eos proteomes (**Figures 2B, C**).

Subsequently, a GSVA was performed ranking the differential abundance and respective p-value of proteins from specific gene sets in IHC15 cells to Eos, to evaluate enrichment. The gene sets analyzed were chosen to cover a broad range of general eosinophilic functions. While the IHC15 cells did not show significant differences to Eos in gene sets representing eosinophils pathway, differentiation, chemotaxis or migration, they were significantly different to Eos in gene sets representing eosinophil-mediated immunity and activation (**Figure 2D**).

To increase the depth of our analysis further and find more specific similarities between the cell line and Eos, we assessed exclusively expressed proteins among the samples by plotting their gene IDs in Venn diagrams without considering abundance. We found that the differentiated cells generally shared more proteins with Eos than the undifferentiated cells, and IL-5 slightly increased the number of exclusively shared proteins with Eos (**Figures 2E, F**, **Supplementary Figure 2A**). A pathway enrichment on the exclusively shared proteins extracted from the Venn diagrams was conducted using Metascape. The goal was to identify shared pathways between differentiated cells (± IL-5) and Eos to validate and expand the results previously observed in the GSVA analysis. The common pathways shared in all cells were hemostasis, bacterial defense, immune response and programmed cell death. Myeloid cell differentiation was only found to be shared between DHC15 and Eos, but not IHC15. In contrast, pathways associated to regulation of cell morphogenesis, adhesion and migration were only found in IHC15 cells and Eos, but not in DHC15 (**Figures 2G, H**).

Lastly, to identify differences between Eos and the cell line cells, we plotted a Venn Diagram simultaneously comparing gene ID lists from HC15, DHC15, IHC15 and Eos (**Supplementary Figure 2A**). In this analysis, 131 proteins could be identified that were expressed exclusively in Eos but not in any of the cell line cells. The gene IDs from these proteins were used in pathway enrichment analysis (**Supplementary Figure 2B**). Of the pathways found as enriched from the proteins exclusively abundant in Eos, several were related to immune response and activation, further confirming the results of the GSVA indicating a difference between the cell line cells and Eos in these gene sets.

In summary, our data indicate that the cell line cells are more similar to Eos than to Neutros and the differentiated cell line cells are more similar to Eos than the undifferentiated cell line cells. Most differences of the cell line cells to Eos were found in immunity and activation pathways.

### 3.2 Differentiated HC15 cells resemble eosinophilic precursors in their morphology, surface marker expression, TF expression and granule protein expression

The proteomics data revealed a high similarity between IL-5-differentiated cells and Eos in overall eosinophilic and differentiation associated proteins. We aimed to evaluate this observation by investigating an array of eosinophilic features, such as morphology and the profiles of surface markers, TFs and granule proteins in detail.

#### 3.2.1 Morphology

**Figure 3:**
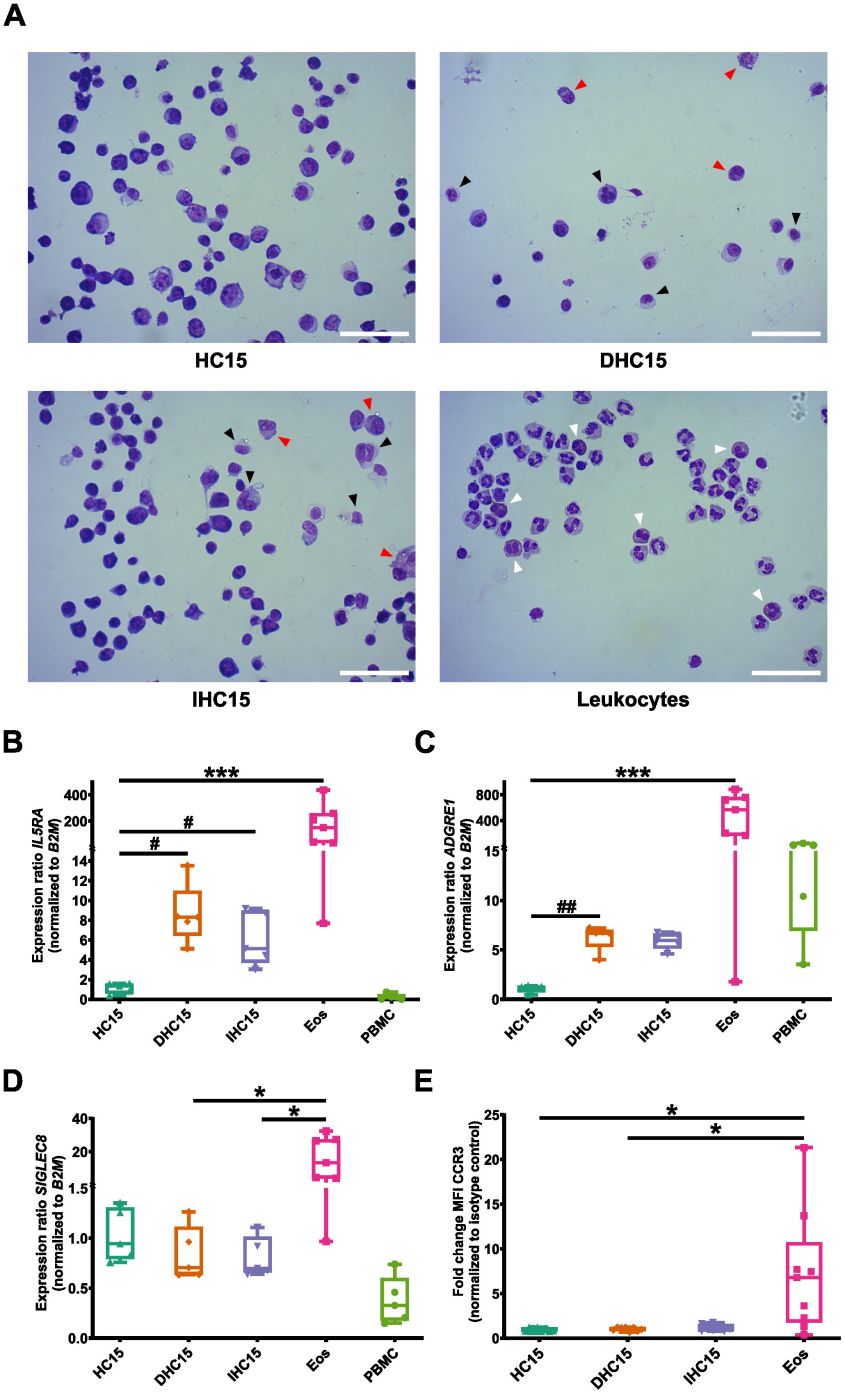
Morphology and surface marker expression of cell line cells and Eos. **(A)** Hemacolor^®^ staining of cell line cells and leukocytes. Black arrows indicate unregular nucleus structure and red arrows indicate high granular content in the differentiated cell line cells. White arrows point to Eos in the leukocytes. Representative images of 5 independent experiments. Scale bar depicts 50 µm. **(B-D)** mRNA expression levels of surface markers relevant in eosinophil biology: **(B)** *IL5RA*, **(C)** *ADGRE1*, **(D)** *SIGLEC8* measured in RT-qPCR (N=5-7). **(E)** Surface exposure of CCR3 measured by flow cytometry (N=8-9). Data is depicted as boxplots. * shows significance vs. Eos, # shows significance vs. HC15. *P<0.05, ***P<0.001; #P<0.05, ##P<0.01; Kruskal-Wallis test with Dunn’s multiple comparisons.

We used hemacolor-stained cytospins to determine the morphology of the differentiated cells. In general, all cell line cells had a different appearance than Eos (marked by white arrows). The cell line cells’ nuclei were round and big, whereas Eos had a characteristic bilobed nucleus structure. Moreover, the cell line cells had a lower proportion of cytosol in comparison to Eos. Additionally, in Eos a deep red granule protein staining in the cytosol could be observed which was not visible in the cell line cells. Differentiation seemed to not visibly change the appearance of the cell line cells. However, some differentiated cells presented with an irregular nucleus shape (black arrows) or a high granule content in the cytosol (red arrows) (**Figure 3A**).

#### 3.2.2 Surface marker expression

Next, we investigated the mRNA expression of four eosinophil surface markers: *IL5RA* (encoding IL-5Rα), *ADGRE1* (encoding EMR1) and *SIGLEC8*. Upon differentiation of the cell line cells with and without IL-5, we found an increase in the mRNA expression of *IL5RA* and *ADGRE1* but not of *SIGLEC8*. However, the mRNA levels of all three receptors remained below the levels in Eos. In PBMCs *IL5RA* mRNA could not be detected. Nonetheless, PBMCs expressed low levels of *SIGLEC8* and high levels of *ADGRE1* (**Figures 3B-D**). Due to a lack of suitable qPCR primers, we assessed CCR3 exposure *via* flow cytometry and found CCR3 exposed on about 1% of the cell line cells, regardless of their differentiation status and on about 7% of Eos (**Figure 3E**, for gating strategy see **Supplementary Figures 3A, B**).

#### 3.2.3 TF expression

A complex interplay between various TFs ensures a controlled and specified gene transcription during differentiation. We studied the mRNA expression of three TFs that are involved in eosinophil differentiation at different stages: *GATA1* (early eosinophil lineage commitment), *ID2* (regulation of end stage differentiation), *SPI1* (encoding PU.1, regulation of granule protein production). PBMCs were added as a control for other cell types.

**Figure 4:**
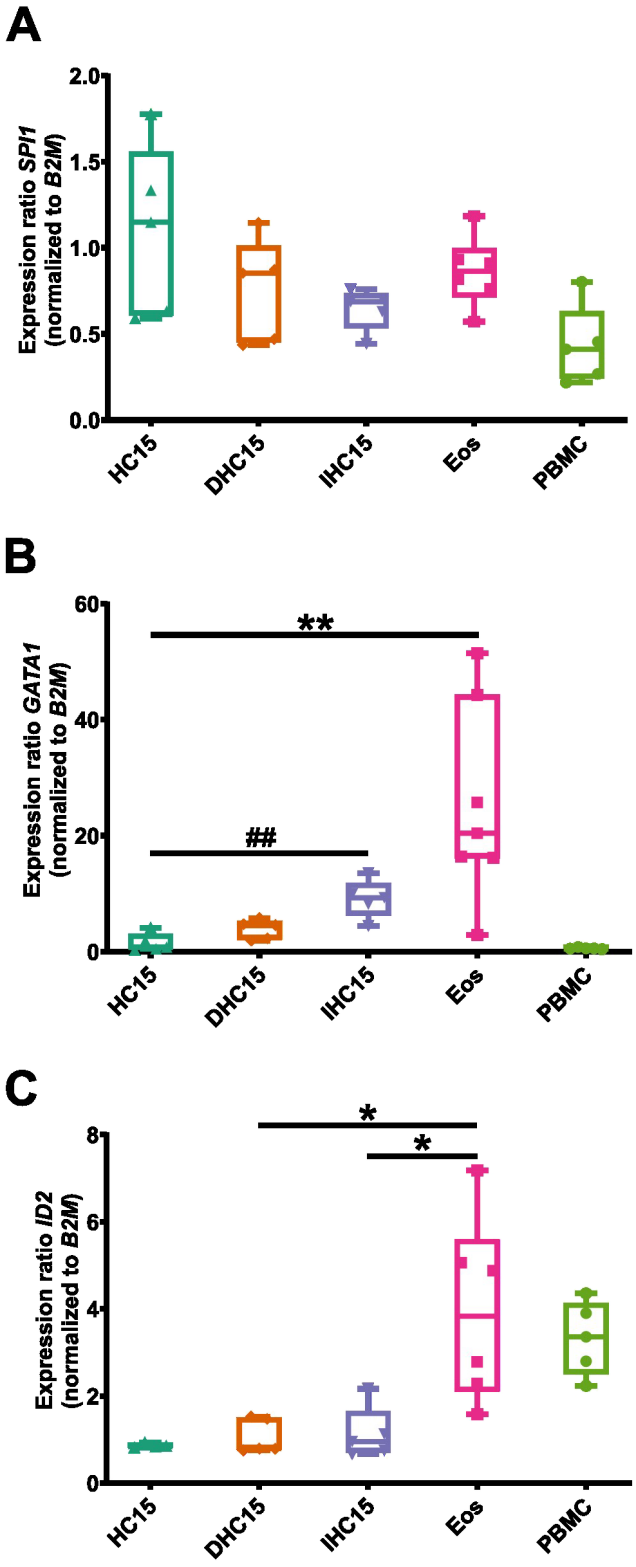
TF profile of the cell line cells and Eos. **(A-C)** mRNA expression levels of TFs relevant to eosinophil differentiation: **(A)** *SPI1*, **(B)** *GATA1* and **(C)** *ID2* (N=5-7). Data is depicted as boxplots. * shows significance vs. Eos, # shows significance vs. HC15. *P<0.05, **P<0.01; ##P<0.01; Kruskal-Wallis test with Dunn’s multiple comparisons.

Overall, the expression levels of *SPI1* were comparable between all cell types. No significant changes in *SPI1* levels were observed (**Figure 4A**). In the case of *GATA1* and *ID2*, Eos showed the highest expression levels. The expression of *GATA1* increased significantly in IL-5-differentiated cells compared to undifferentiated cells (**Figure 4B**). In contrast, the expression of *ID2* in the cell line cells remained unchanged upon differentiation (**Figure 4C**). In PBMCs, no *GATA1* expression was detected whereas the expression of *ID2* was comparable to that in Eos.

#### 3.2.4 Granule protein profile

An important characteristic of eosinophils is their high content of cationic granule proteins, which are cytotoxic and released upon activation. We studied EMBP and EPX, two prominent representatives of this protein family.

**Figure 5:**
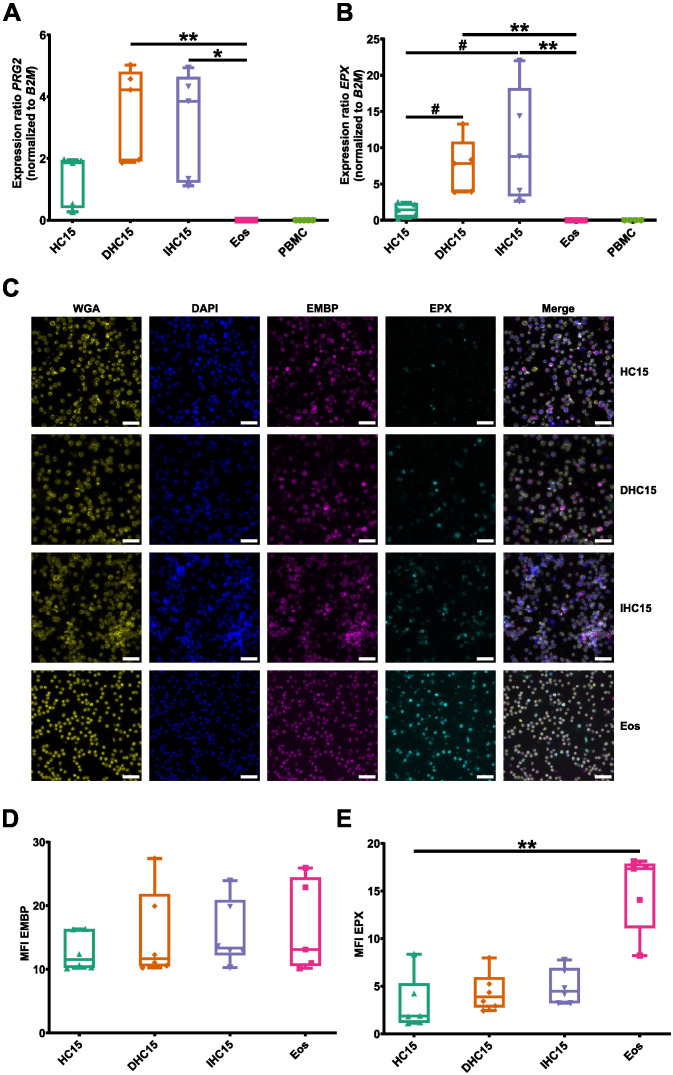
Differentiation induces granule protein expression in HC15 cells on a mRNA level. **(A, B)** mRNA expression levels of eosinophil-specific granule proteins: **(A)** *PRG2* and **(B)** *EPX* (N=5-7). **(C-E)** Granule protein levels. **(C)** Granule proteins are stained in red (EMBP) and green (EPX), respectively. DAPI (blue) and wheat germ agglutinin (yellow) were used to identify cell nuclei and cell borders, respectively. Representative images of 6 independent experiments. Scale bar depicts 50 µm. **(D, E)** Quantification of granule protein levels **(D)** EMBP and **(E)** EPX. 5 pictures of randomly chosen areas were analyzed. The mean fluorescence intensity per cell was determined using ImageJ. Data is depicted as boxplots. * shows significance vs. Eos, # shows significance vs. HC15. *P<0.05, **P<0.01; #P<0.05; Kruskal-Wallis test with Dunn’s multiple comparisons. MFI, mean fluorescent intensity.

On a mRNA level, neither *EPX* nor *PRG2* (encoding EMBP) could be detected in Eos or PBMCs. However, mRNA of both proteins was detected in HC15 cells, and differentiation with or without IL-5 increased the levels to a significantly higher amount than Eos (**Figures 5A, B**). The levels of both proteins were additionally determined using immunofluorescent staining techniques. EMBP and EPX were found in the cytosols of all cell line cells and Eos (**Figure 5C**). The levels of EPX were higher in Eos than in the cell line cells, while the EMBP levels were similar in all cells. Differentiation did not affect the granule protein levels (**Figures 5D, E**).

In summary, the cell line cells presented with a different morphology than Eos. Differentiation induced an increase in *IL-5R*, *EMR1* and *GATA-1* expression, as well as an overexpression of EMBP (on the mRNA level) and EPX (on the mRNA and protein level) compared to Eos on an mRNA level but not on a protein.

### 3.3 The HC15 cell line resembles eosinophils in its PLT-dependent and -independent migration and adhesion

According to our proteomics data, IL-5-differentiated cells showed a high similarity to Eos in regards to chemotaxis and migration. By studying aspects of cell migration individually, we aimed to get a clearer picture of how the cell line cells migrate compared to Eos.

**Figure 6:**
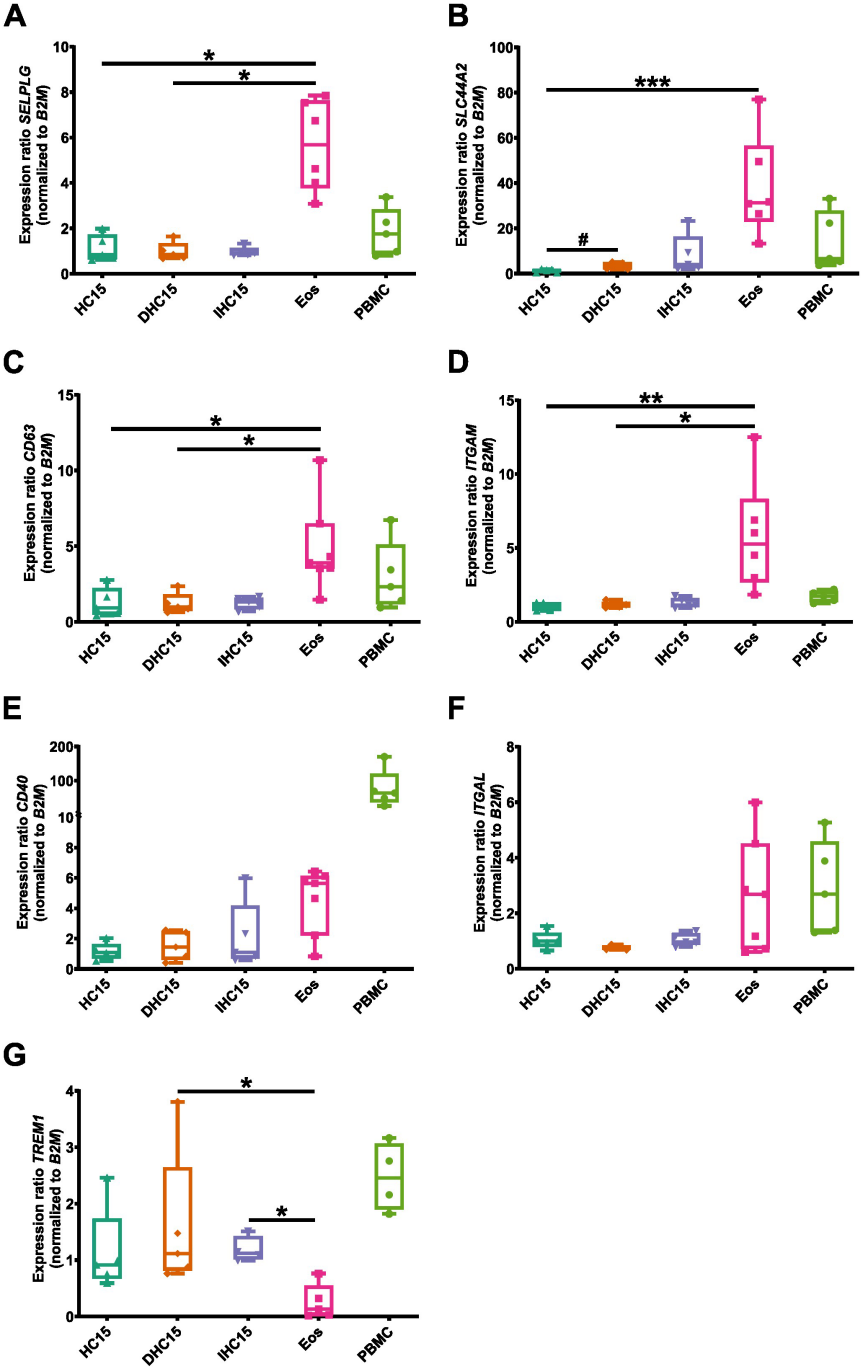
mRNA levels of various adhesion markers in cell line cells and Eos. mRNA expression levels of relevant adhesion markers: **(A)** *SELPLG*, **(B)** *SLC44A2*, **(C)** *CD63*, **(D)** *ITGAM*, **(E)** *CD40*, **(F)** *ITGAL*, **(G)** *TREM1*) (N=5-7). Data is depicted as boxplots. * shows significance vs. Eos, # shows significance vs. HC15. *P<0.05, **P<0.01, ***P<0.01; #P<0.05; Kruskal-Wallis test with Dunn’s multiple comparisons.

First, we determined the mRNA expression levels of an array of important adhesion markers, such as *SEPLG* (encoding PSGL-1), *SLC44A2*, *CD63*, *ITGAM* (encoding CD11b), *CD40, ITGAL* (encoding CD11a) and *TREM1* (46). Most adhesion markers (*SELPLG* (**Figure 6A**), *SLC44A2* (**Figure 6B**), *CD63* (**Figure 6C**) and *ITGAM* (**Figure 6D**) were expressed significantly higher in Eos than in the cell line cells. *CD40* (**Figure 6E**) and *ITGAL* (**Figure 6F**) showed a trend towards higher expression in Eos, whereas *TREM1* (**Figure 6G**) showed lower expression in Eos compared to the cell line cells. Moreover, differentiation induced a significant increase in the expression of *SLC44A2* in the cell line cells. A non-significant trend towards increased expression in DHC15 and IHC15 cells, respectively compared to HC15 cells was also found in *CD40* and *SLC44A2*. In PBMCs, high levels of *CD40* compared to all other cell types were found, while the expression of *ITGAL* in PBMCs was comparable to Eos. All other markers were expressed in PBMCs at a similar level as in the cell line cells.

**Figure 7:**
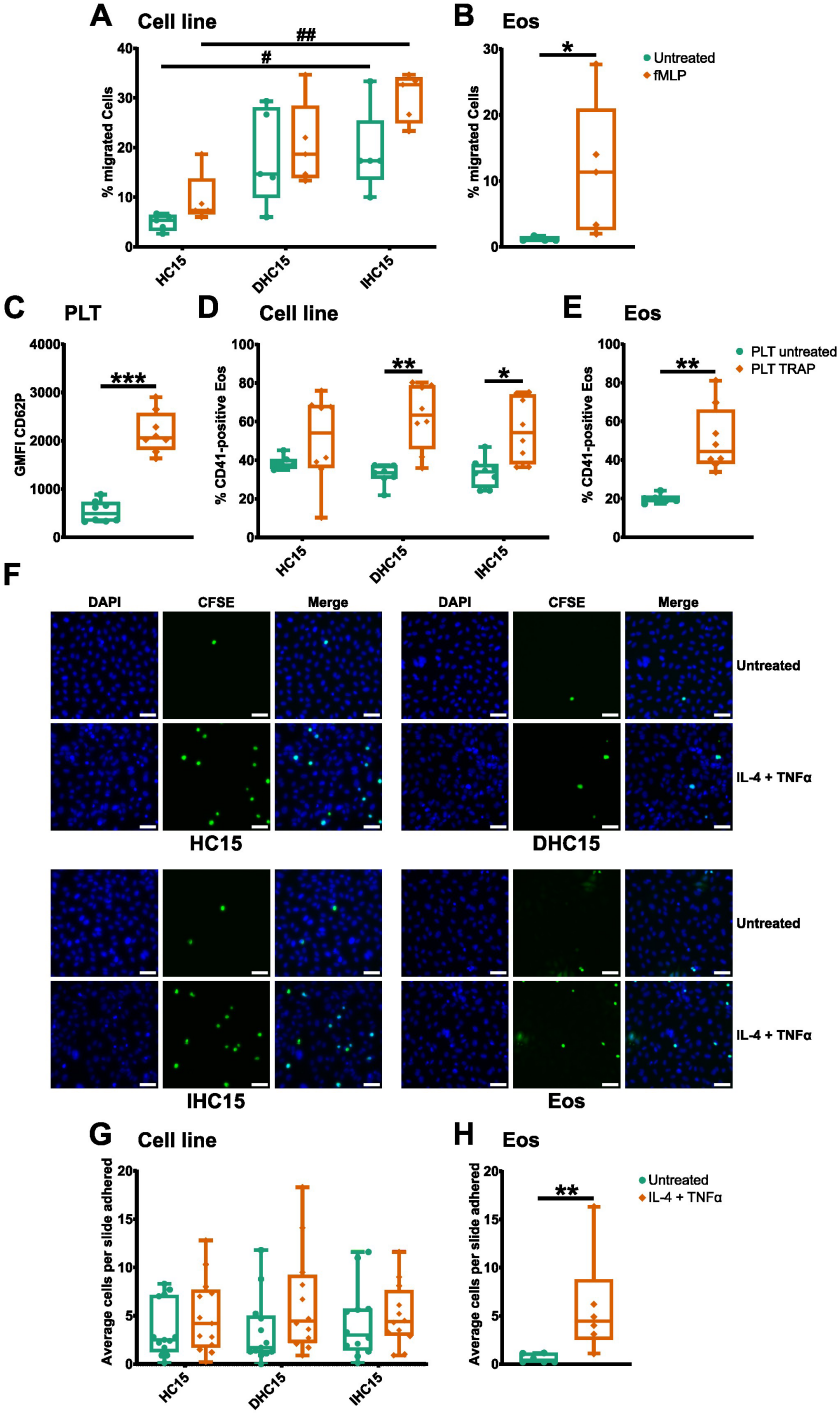
IL-5-differentiated cells have a similar capacity to migrate, adhere and aggregate as Eos. **(A, B)** Transwell cell migration assay. After stimulation with 100 nM fMLP for 60 min, cells in the lower chamber were counted in a hemocytometer (N=5). **(A)** Cell line migration. **(B)** Eos migration. **(C-E)** Aggregate formation assay with PLTs. Resting or activated PLTs were incubated with cells in a 200:1 ratio and aggregate formation was analyzed by flow cytometry (N=8). **(C)** PLT activation was confirmed by measurement of CD62P expression. **(D)** PLT-cell line aggregate formation. **(E)** PLT-Eos aggregate formation. **(F-H)** Cell adherence to HUVECs in a PLT-rich environment. CFSE-stained cell line cells or Eos were incubated with resting or activated HUVECs with PLTs for 30 min and fixed for immunofluorescence analysis. **(F)** DAPI (blue) was used to identify the nuclei and CFSE (green) was used to identify eosinophilic cells. Representative images of 6–13 independent experiments. Scale bar depicts 50 µm. 10 pictures of randomly chosen areas were analyzed. **(G)** Quantification of cell line adherence to HUVECs. **(H)** Quantification of Eos adherence to HUVECs. Data is depicted as boxplots. * shows significance between treatments, # shows significance between differentiations. *P<0.05, **P<0.01, ***P<0.001; #P<0.05, ##P<0.01; Two-way ANOVA with Tukey’s multiple comparisons **(A, D, G)**, Mann-Whitney test **(B, C, E, H)**.

Next, we evaluated the cell lines’ ability to migrate towards the chemotactic stimulus fMLP using a transwell assay. Our results show that differentiation increased unspecific cell migration which was generally higher in the cell line cells than in Eos. Furthermore, in Eos, a significantly increased migration could be observed after the addition of a chemotactic stimulus. In IHC15 cells, a similar, but non-significant trend could be observed. (**Figures 7A, B**).

To study the influence PLTs might have on migration of the cell line cells, we analyzed the interaction between the cells and PLTs with and without PLT stimulation (see **Supplementary Figure 4A** for gating strategy of platelet stimulation). All cell line cells showed a similar baseline PLT binding capacity. Without stimulation, about 40% of the cell line cells were bound to PLTs after 15 min of incubation, whereas 20% of Eos bound PLTs. Activation of PLTs significantly increased the capacity of binding to the differentiated cell line cells and Eos to a similar extend. A similar trend was visible in the HC15 cells, which was however not significant (**Figures 7C-E**, for gating strategy see **Supplementary Figure 4B**).

Moreover, the adherence of the cells to stimulated HUVECs was tested. Since it is known that PLTs assist this process in a physiological setting, this experiment was performed with and without the addition of PLTs. Overall, more eosinophilic cells adhered in a PLT-rich setting than without PLTs (data not shown). In a PLT-rich environment, stimulation of HUVECs tended to increase the binding capacity of all cells. However, this was only significant in the case of Eos. Hence, our data indicate that the cell line cells overall, but cells differentiated without IL-5, specifically have a similar ability to adhere to HUVECs as Eos (**Figures 7F-H**).

Lastly, to investigate the influence of different adhesion receptors on the migratory functions of the cell line cells, we repeated the all chemotaxis and migration assays while blocking the adhesion markers studied before (see **Figure 6**). Since we did not find CD40 to be present on the surface of Eos (data not shown), we did not include its blocking into our experiments. However not significant, the blocking of SLC44A2, CD11b and TREM-1 lead to a slight reduction of Eos migration. This trend could also be seen in the undifferentiated cell line cells (**Supplementary Figures 5A, B**). Furthermore, PLT-aggregate formation was found to be strongly dependent on PSGL-1, as the blocking of this receptor led to a strong reduction of PLT binding in all cell types (**Supplementary Figures 5C-E**). In the case of cell adherence to stimulated HUVECs in a PLT-rich environment, blocking of the adherence receptors seemed to have little to no effect (**Supplementary Figures 5F, G**).

In summary, the cell line cells presented with a different surface marker expression than Eos. Nonetheless, the differentiated cell line cells showed a similar profile of chemotaxis, PSGL-1-dependent PLT-aggregate formation and PLT-dependent adherence as Eos.

### 3.4 Differentiated cell line cells show a different response to activation than eosinophils

After analyzing gene sets that were similar between IL-5-differentiated cells and Eos in our proteomics data, we set to investigate the differing gene sets, in particular activation and immunity. Eosinophil activation involves the increased exposure of adhesion markers, as well as the release of inflammatory mediators and granule proteins by degranulation (19). We activated the cells with the protein kinase C activating compound PMA to observe the exposure of the adhesion receptors CD11b and CD63 and the secretion of CCL5 and EPX (47).

**Figure 8:**
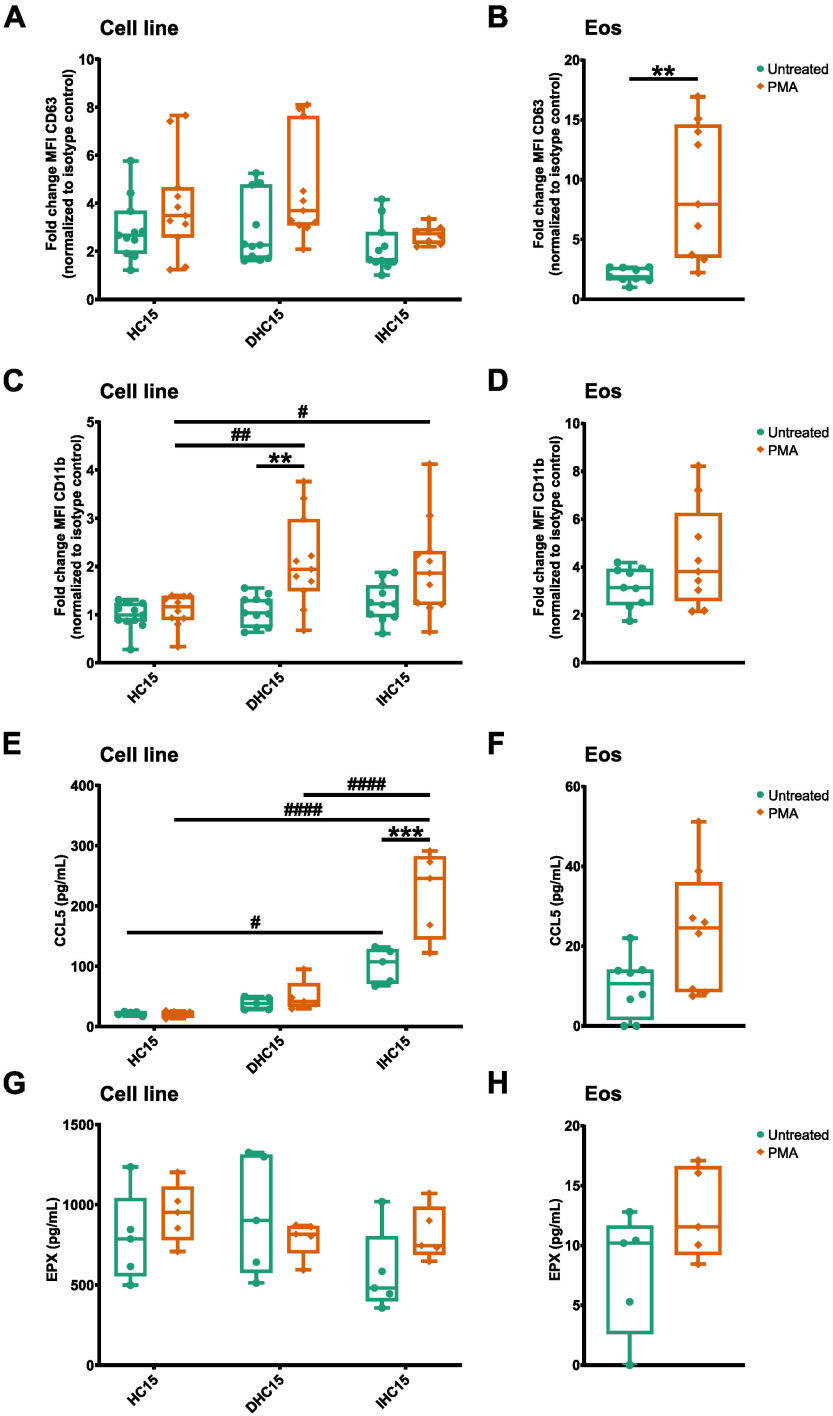
IL-5-differentiated cells heterogenously respond to activation compared to Eos. Cells were activated with 10 µM PMA for 90 min. **(A-D)** Surface marker levels measured by flow cytometry (N=11). **(A)** CD63 levels on cell line cells. **(B)** CD63 levels on Eos. **(C)** CD11b levels on cell line cells. **(D)** CD11b levels on Eos. **(E-H)** Cytokine and granule protein release measured by ELISA (N=5-8). **(E)** CCL5 secretion from cell line cells. **(F)** CCL-5 secretion from Eos. **(G)** EPX secretion from cell line cells. **(H)** EPX secretion from Eos. Data is depicted as boxplots. * shows significance between treatments, # shows significance between differentiations. **P<0.01, ***P<0.001; #P<0.05, ##P<0.01, ####P<0.0001; Two-way ANOVA with Tukey’s multiple comparisons **(A, C, E, G)**, Mann-Whitney test **(B, D, F, H)**.

In the cell line cells, PMA activation did not induce significant changes in CD63 exposure. However, in DHC15 cells, PMA induced an exposure of CD11b which could also be seen as a trend in IHC15 cells. In contrast, in Eos, PMA activation induced a significant increase of CD63 but not of CD11b (**Figures 8A-D**, for gating strategy see **Supplementary Figures 6A-C**). IHC15 cells showed a significantly enhanced CCL-5 secretion in response to PMA, a trend which could also be seen in Eos. Nonetheless, the total amount of CCL5 secreted from the IHC15 cells was about ten times higher than from that Eos. Furthermore, while the cell line cells showed an about ten times higher baseline secretion of EPX than Eos (**Figure 8E**), the EPX secretion was not affected by PMA in any of the cells (**Figure 8H**).

In summary, the differentiated cell line cells showed a different response to PMA-induced activation than Eos. While the surface marker exposure on the cell line cells in response to PMA was less pronounced and to some extend opposite to that on Eos, the baseline granule protein secretion from all cell line cells and the PMA-induced secretion of CCL5 from differentiated cells was increased compared to Eos.

### 3.5 The unstimulated inflammatory milieu released by differentiated eosinophilic cells features chemokines that are important for the recruitment of neutrophils and T-cells

**Figure 9:**
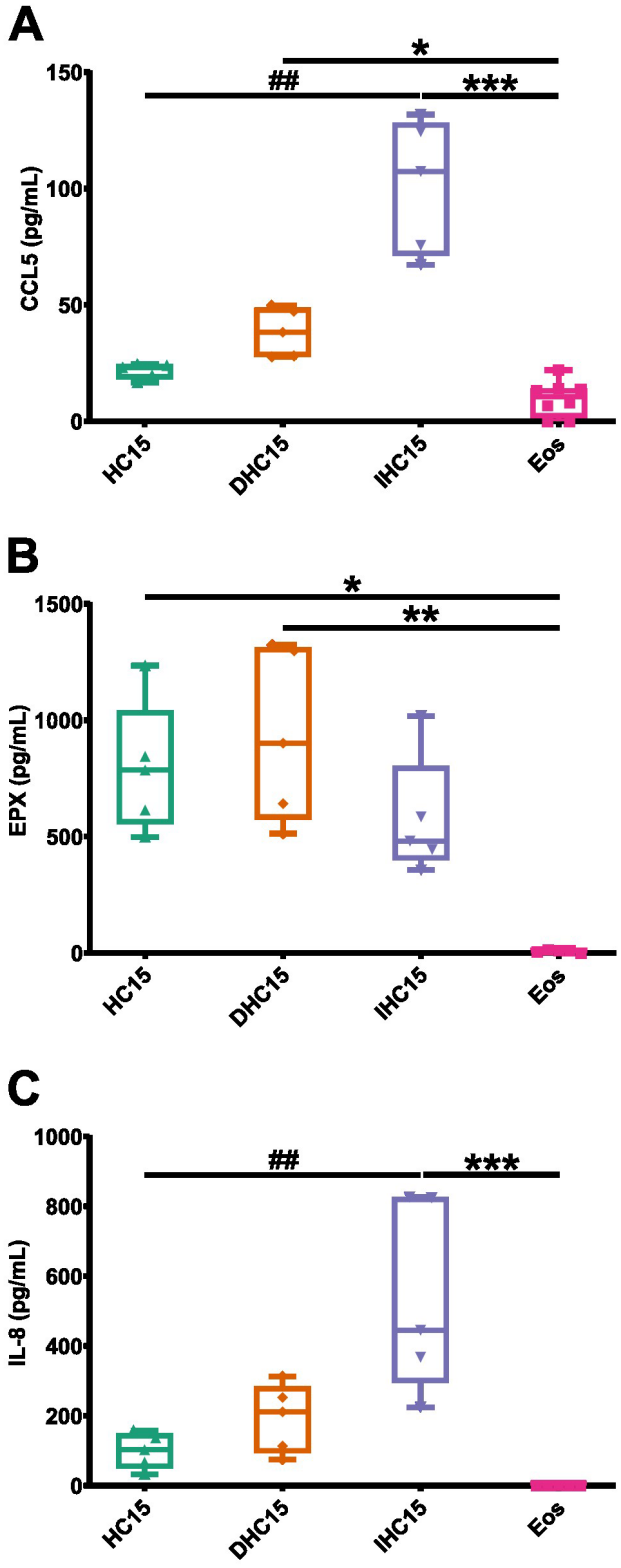
IL-5-differentiated cells produce a granulocyte- and T-cell-attracting inflammatory milieu. Cytokine- and granule protein-secretion profiles from unstimulated cell line cells and Eos measured by ELISA (N=5-8): **(A)** CCL-5, **(B)** EPX, **(C)** IL-8. Data is depicted as boxplots. * shows significance vs. Eos, # shows significance vs. HC15. *P<0.05, **P<0.01, ***P<0.01; ##P<0.01; Kruskal-Wallis test with Dunn’s multiple comparisons.

Our proteomics data suggest that IL-5-differentiated cells might differ from Eos in proteins associated with eosinophil-mediated immunity. We compared the CCL-5 and EPX release between the cell line cells and Eos (data from experiment depicted in **Figures 8E-H**). Indeed, in line with the proteomics data the CCL5 and EPX release was significantly increased in the cell line cells compared to Eos (**Figures 9A, B**).

We therefore investigated the secretion profiles of other cytokines of different functionality. We chose an array of cytokines involved in Th1, Th2, proinflammatory and anti-inflammatory signaling or granulocyte/eosinophil related pathways. Of the tested cytokines, only IL-8 could be detected in the supernatants, which showed a similar secretion profile as CCL5 (**Figures 9A-C**, **Supplementary Figure 7**).

In summary, our data indicate that the cytokine profiles of cell line cells significantly differ from those of Eos, with cell line cells having a significantly higher EPX release and the differentiated cell line cells having a significantly higher IL-8 and CCL-5 release.

## 4 Discussion

**Figure 10:**
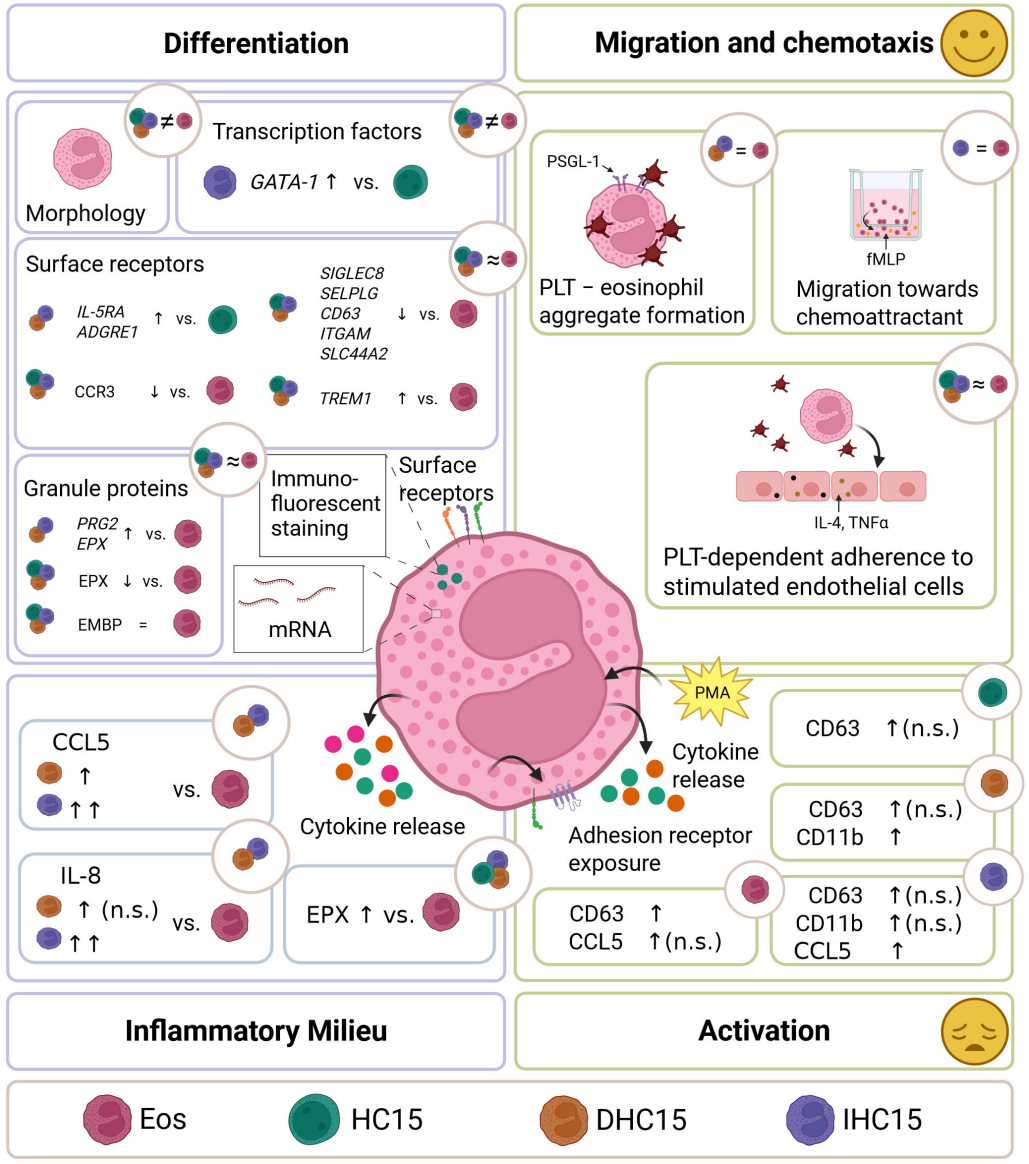
Summary. Cell line cells and Eos were compared in differentiation, inflammatory milieu, activation and migration and chemotaxis. Morphology, granule protein levels and RNA expression profiles indicate a precursor state of the differentiated cell line cells. Migration and chemotaxis patterns with or without PLTs were similar between differentiated cell line cells and Eos. Cell line cells showed a more heterogenous activation response than Eos. Without activation, differentiated cell line cells induced a neutrophil- and T-cell-attracting inflammatory milieu. Addition of IL-5 during differentiation enhanced some of the inflammatory milieu induction and the specificity of migration, but had minor effects on differentiation.

In this work, the HC15 cell line was characterized and compared to Eos in the aspects of differentiation, inflammatory milieu, activation, as well as migration and chemotaxis (see **Figure 10** for a graphical summary of all investigations). The proteomes of the differentiated cell line cells and Eos showed similarities in differentiation pathways. However, differences in their morphology, granule protein levels and mRNA expression profiles suggest that the cell line remains in an eosinophil precursor state after differentiation. The cell line cells behaved similarly to Eos regarding migration and chemotaxis. They showed specific migration towards a chemotactic stimulus and similar adherence patterns to PLTs and HUVECs in a PLT-rich environment. The cell line cells’ response to activation differed from that of Eos. Moreover, differentiated cell line cells induced a neutrophil- and T-cell-attracting inflammatory milieu by secreting CCL5 and IL-8, which Eos did not. The addition of IL-5 during differentiation enhanced some of the described effects, such as the inflammatory milieu induction and the specificity of migration, but had only minor effects on differentiation.

The use of the HC15 cell line as a model system for eosinophils can be beneficial in some settings, however it also has limitations. When the cell line was first described, a continuous culture at a slightly alkaline pH was determined essential for eosinophil differentiation of the otherwise neutrophil-prone precursor cell line (30). Our proteomics data indicate that the phenotype of HC15 cells kept at pH 7.6-7.8 resembled an eosinophilic phenotype rather than a neutrophilic phenotype after differentiation. Furthermore, the TOST-test, a statistical analysis evaluating the similarity of two samples, revealed that the differentiated cell line cells were significantly similar to Eos.

Nonetheless, the overall conformity of a large set of proteins might hide meaningful differences in a smaller subset of proteins within. Accordingly, the cell line cells and Eos were morphologically different, whereas differentiation-related pathways were significantly similar in the GSVA analysis. Furthermore, differentiation did not drastically change the appearance of the cell line cells. We therefore conclude that in contrast to what has been published before, morphology alone might not be well-suited to evaluate the differentiation status of the HC15 cell line (29–31, 33, 35, 36, 38). Since the evaluation of morphologic features can also be subjective, difficult to automatize and is therefore prone to misjudgment, we investigated other cost- and labor-effective methods to assess differentiation.

Of the four tested eosinophil-specific surface markers, only CCR3 could be stained successfully for flow cytometry. All receptors that were not detected by flow cytometry, were studied on an mRNA level instead. In a previous study, CCR3 was found to be expressed constitutively on freshly isolated eosinophils (48). In contrast, we measured CCR3 on less than 10% of Eos and only marginally on the cell line cells.

Several findings indicate that the cell line cells resemble an eosinophilic precursor state rather than mature eosinophils even after differentiation. The expression levels of *IL5RA* and *ADGRE*, genes encoding receptors highly specific for eosinophils (49, 50), were increased in the cell line cells upon differentiation. Nonetheless, their levels remained below those of primary cells. *SICLEC8*, a gene encoding a late maturation marker, was not expressed by the HC15 cell line. In line with that, other eosinophilic precursor cell lines such as Eol-1 and AML14.3D10 have been reported not to express *SICLEC8* (51). In a previous study investigating the levels of several TFs in human eosinophilic precursors and mature eosinophils, low levels of GATA-1 in the common myeloid precursor were observed, which increased through differentiation to a maximum in mature eosinophils (2). ID2 on the other hand is known to be produced late during eosinophil maturation (3). In line with a precursor profile, the expression of *GATA1* was induced by IL-5 differentiation in our experiments, but remained much lower than in Eos, whereas the expression of *ID2* remained low in the cell line cells regardless of their differentiation status. While not being essential for granule protein production, PU.1 strongly enhanced the expression of MBP in murine fibroblasts (4). In our experiments, mRNA levels of both, *EPX* and *PRG2* (encoding EMBP) were increased after differentiation. Therefore, the low expression levels of *SPI1* (encoding PU.1) in the HC15 cell line compared to Eos in our experiments were unexpected. Nonetheless, the role of PU.1 in eosinophil differentiation is still not completely understood and other factors involved in granule protein expression might explain our findings.

Our proteomics data suggested a high resemblance of the HC15 cell line and Eos proteomes in migration- and chemotaxis-related pathways. Therefore, we investigated a variety of chemotactic features in detail. *In vivo*, leukocyte extravasation involves a series of steps and several types of cells. Activated PLTs bind to the endothelial surface and flag sites of inflammation. They interact with rolling leukocytes, which leads to their arrest and assists leukocyte extravasation (24). Moreover, eosinophil-PLT aggregates regularly occur in the circulating blood in allergy (52). To assess several aspects of physiological migration, we studied isolated migration towards a chemotactic stimulus, the formation of aggregates with PLTs and adherence to HUVECs in a PLT-rich environment.

*In vivo*, most eosinophils fully mature in the bone marrow and gain their ability to migrate at a late differentiation state (3). The cell line cells showed high similarities to Eos in all aspects of migration. In our experiments, specific cell migration and adherence, as well as PTL binding were already visible in undifferentiated cells and stayed largely unaffected by the differentiation. This is in accordance with previous works investigating migration using this cell line (33, 53).

When blocking different adherence receptors on the cell line cells or Eos, we found PSGL-1 to be highly relevant for the formation of aggregates with activated PLTs. This fits previous studies from other groups, as PSGL-1 is a well-established ligand for the platelet activation marker P-selectin (54). Moreover, PSGL-1/P-selectin axis has been identified as an important mediator between leukocytes and platelets in several inflammatory settings, including allergy (55, 56). The blocking of adherence receptors on the cell line cells or Eos during chemotactic migration or adherence assays only led to slight trends towards reduction. The studied processes might therefore be a result of a complex interaction between several of the studied receptors or include other known or unknown adherence markers.

Of note, almost all tested adherence or adherence-related markers (*SELPLG*, *CD40*, *CD63*, *ITGAL*, *ITGAM* and *SLC44A2*) were expressed at lower levels in the cell line cells than in Eos. The only receptor that showed increased expression in the HC15 cell line was TREM-1, known for its role in amplifying inflammatory responses. TREM-1 enhanced the chemotaxis of murine neutrophils in response to different inflammatory stimuli even though its binding partners are still largely unknown (57). Overexpression of TREM-1 specifically in differentiated cells might explain the increased unspecific migration of the differentiated cells compared to Eos. We suppose that the HC15 cell line is a useful tool to study leukocyte chemotaxis without the need to differentiate the cells before use. Nonetheless, the assumed precursor state might set limits to some functional experiments related to chemotaxis.

The investigation of surface marker expression and granule secretion revealed similarities and differences between the HC15 cell line and Eos in response to activation. Most prominently, the EPX secretion was not affected by PMA in the HC15 cell line, whereas although not significant, a trend towards PMA-induced EPX secretion was visible in Eos. For CCL-5 to be secreted from the cell line cells, IL-5 addition during differentiation seemed to be essential. On the other hand, the exposure of CD63 and CD11b was most clearly induced by PMA in cells differentiated without IL-5. Our results from the proteomics data are in line with the activation experiments.

We were intrigued by the high secretion of CCL-5 from IL-5-differentiated cells even without PMA-activation. We therefore measured the secretion of more cytokines specific for various inflammatory responses and found IL-8 to be secreted by resting IHC15 cells as well. CCL5 and IL-8 are involved in the regulation of T-cell- and neutrophil-migration (58, 59). Released mostly by Th2 cells, IL-5 plays a critical role in eosinophil activation (60). Therefore, one explanation for the results found in our experiments could be that IL-5 not only affects differentiation, but also induced a state of mild activation. Eosinophils are known to store an array of cytokines in their granules and secretory vesicles which are released upon activation (61). However, in our experiments, we did not detect the secretion of further cytokines from resting or activated Eos or cell line cells.

For some research questions, the use of cell lines is not suited and therefore, the isolation of primary eosinophils is needed. As mentioned before, this process can be challenging due to technical limitations, such as low purity, spontaneous activation, low yield, high time of labor and high cost. In this work, we used a new protocol to isolate eosinophils from young healthy donors combining the Straightfrom^®^ Whole blood PBMC isolation kit with the Eosinophil isolation kit from Miltenyi. In our hands, this protocol yielded a better recovery of non-activated eosinophils than the current gold standard, the MACSxpress^®^ Eosinophil isolation kit (62). The time of labor, cost, and purity of the retracted Eos were comparable in both isolation protocols. A continued improvement and innovation of eosinophil isolation techniques will help make eosinophil research more accessible and valuable.

In conclusion, this study characterized the features and biological functions of the eosinophilic cell line HC15 in detail. While overall, the differentiated cell line cells showed similarities to Eos especially in chemotaxis-related aspects, there were still differences in some distinct areas, such as morphology, activation and immunity. Adding IL-5 to the differentiation protocol slightly increased the specificity of chemotaxis and induced the secretion of a T-cell and neutrophil attracting inflammatory milieu. In summary, we suppose that the HC15 cell line differentiated towards an eosinophil resembling phenotype is not well-suited for research questions addressing eosinophil activation and immunity, but can be used to investigate migration and chemotaxis, as well as adherence to other cells, such as PLTs.

## Data Availability

The datasets presented in this study can be found in online repositories. The names of the repository/repositories and accession number(s) can be found below: PXD057747 (PRIDE, 63).

## Glossary

IL-5Rα: IL-5 receptor chain α
GATA-1: GATA-binding protein 1
TF: transcription factor
ID2: inhibitor of DNA binding 2
CCR3: C-C chemokine receptor 3
Siglec-8: sialic acid binding Ig like lectin 8
EMR1: EGF-like module-containing mucin-like hormone receptor-like 1 (encoded by *ADGRE1*)
PLT: platelet
HC15: HL-60 clone 15
SB: sodium butyrate
EMBP: eosinophil major basic protein (encoded by *PRG2*)
Eos: primary eosinophil
DHC15: HC15 differentiated with SB
IHC15: HC15 differentiated with SB and IL-5, PBMC, peripheral blood mononuclear cell
Neutro: primary neutrophil
TOST: two one-sided t-test
PCA: principal component analysis
DAP: differentially abundant protein
GSVA: gene set variation analysis
RT-qPCR: Reverse transcription quantitative polymerase chain reaction
*B2M*: beta-2-microglobulin, EPX, Eosinophil peroxidase
CD11a: integrin subunit alpha L (encoded by *ITGAL*)
CD11b: integrin subunit alpha M (encoded by *ITGAM*)
PSGL-1: selectin P ligand (encoded by *SELPLG*)
*SLC44A2*: Solute carier family 44 member 2, PU.1, Spi-1 proto-oncogene (encoded by *SPI1*)
*TREM1*: Triggering receptor expressed on myeloid cells 1
fMLP: N-formyl-Met-Leu-Phe
TRAP: thrombin receptor activator peptide 6
HUVEC: human umbilical vein endothelial cell
CFSE: carboxy fluorescein succinimidyl ester
ELISA: enzyme-linked immunosorbent assay
CCL11: C-C chemokine 11/Eotaxin-1
CCL5: CC chemokine 5/RANTES, regulated on activation, normal T-cell expressed and secreted
ANOVA: analysis of variance
MFI: mean fluorescence intensity.
