# Supplementary material for: The HL-60 clone 15 cell line as a model for leukocyte migration–possibilities and limitations
Source: Front Immunol. 2025 May 27;16:1515993. doi: 10.3389/fimmu.2025.1515993 (PMC12148902; doi:10.3389/fimmu.2025.1515993)
Supplement: Supplementary file 1 [file DataSheet1.pdf]

Supplementary material

Supplementary figures

Supplementary figure 1: Quality control of Eos isolation measured *via* flow cytometry.

Supplementary figure 2: Analysis of proteins shared between cell line cells and Eos.

Supplementary figure 3: Gating strategy for determination of CCR3 levels on eosinophilic cells.

Supplementary figure 4: Gating strategy of aggregate formation assay with PLTs.

Supplementary figure 5: PLT-eosinophil aggregate formation is significantly dependent on PSGL-1 expression on eosinophilic cells.

Supplementary figure 6: Gating strategy for determintation of CD63 and CD11b levels on eosinophilic cells activated with PMA.

Supplementary figure 7: Inflammatory milieu of untreated eosinophilic cells.

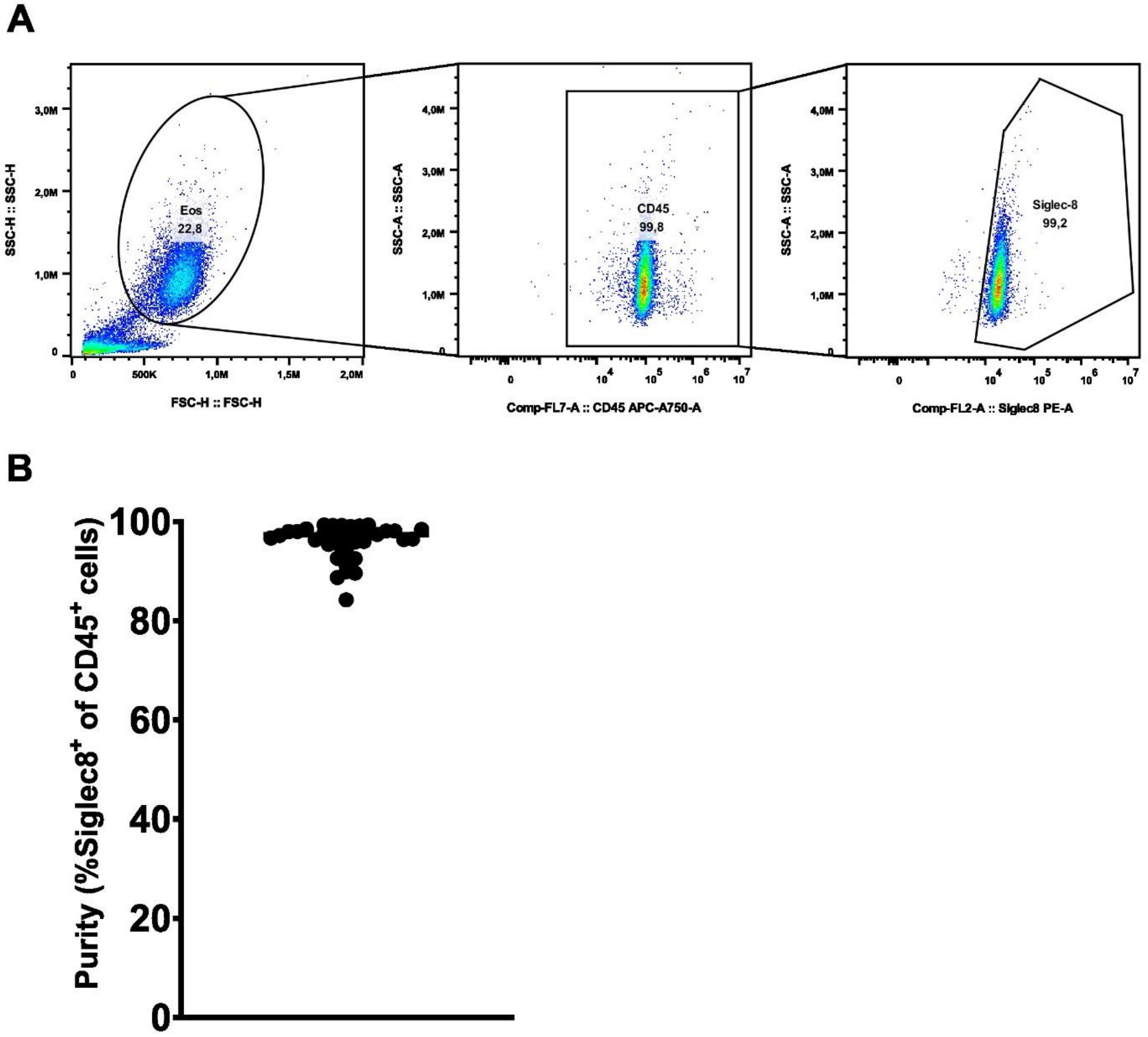

Supplementary figure 1: Quality control of Eos isolation measured *via* flow cytometry. **(A)** Gating strategy. Siglec-8-positive $CD45^+$ cells were determined as Eos. **(B)** Eos (= $CD45^+$Siglec-8^+) purity was 97.3 % [94.8 % - 97.2%] over all experiments (N=38). Data are median with 95% CI.

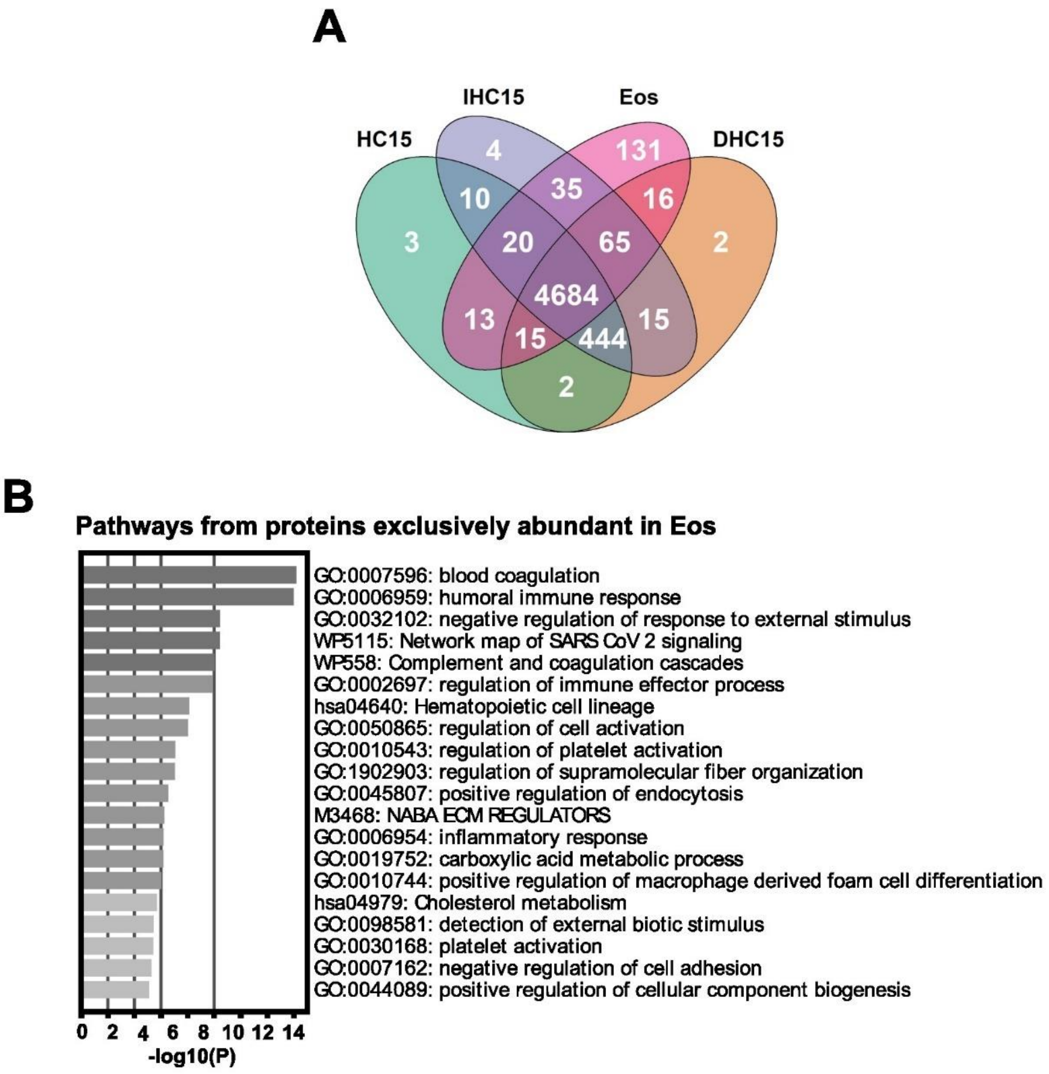

Supplementary figure 2: Analysis of proteins shared between cell line cells and Eos. (A) Venn diagram of proteins shared between cell line cells and Eos. The gene IDs of proteins found in HC15 vs. DHC15 vs. IHC15 vs. Eos were plotted and visualized in a Venn diagram using R version 4.3.3. **(B)** Pathway enrichment analysis of proteins exclusively abundant in Eos. The gene IDs of proteins exclusively abundant in Eos were extracted from the Venn diagram in (A) and a pathway enrichment analysis was computed using Metascape.

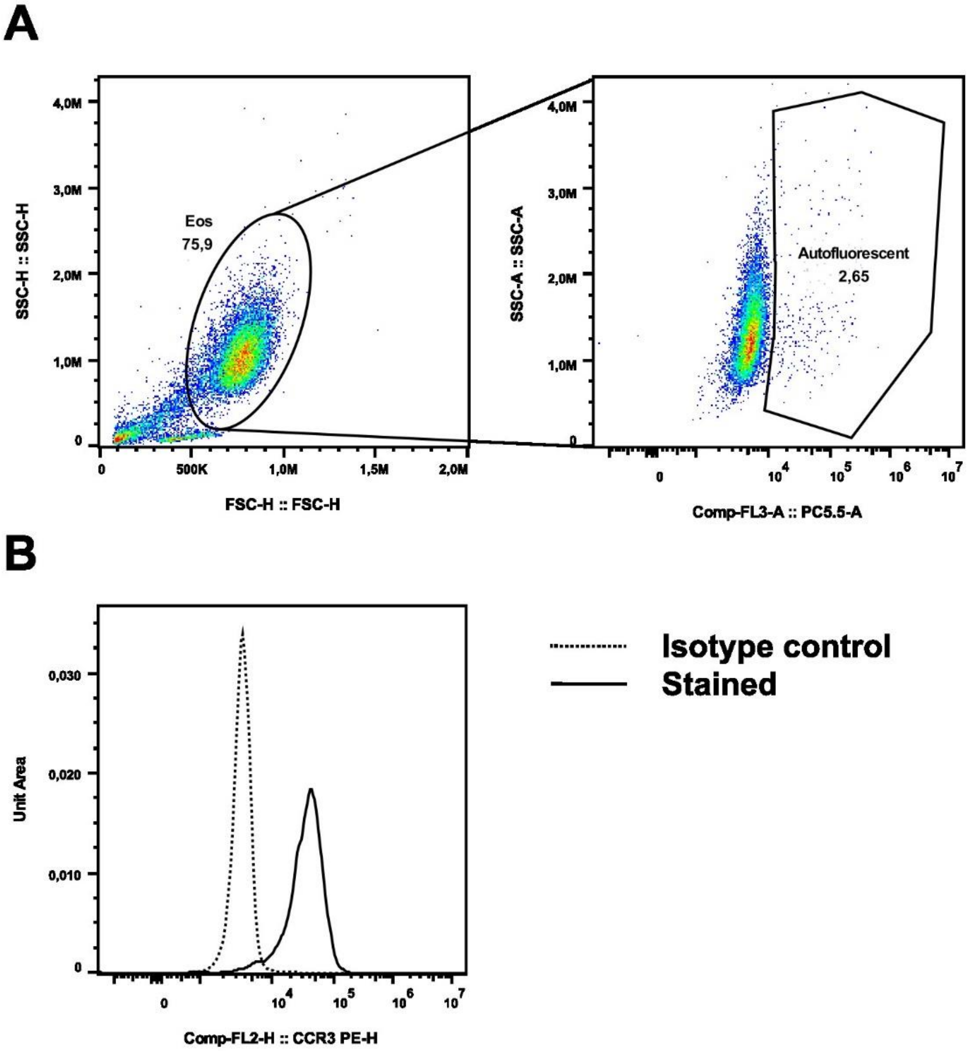

Supplementary figure 3: Gating strategy for determination of CCR3 levels on eosinophilic cells. (A) Eosinophilic cells were identified by size and granularity. Autofluorescent cells were gated out. **(B)** The surface marker levels of CCR3 were determined by a MFI-shift of the respective marker.

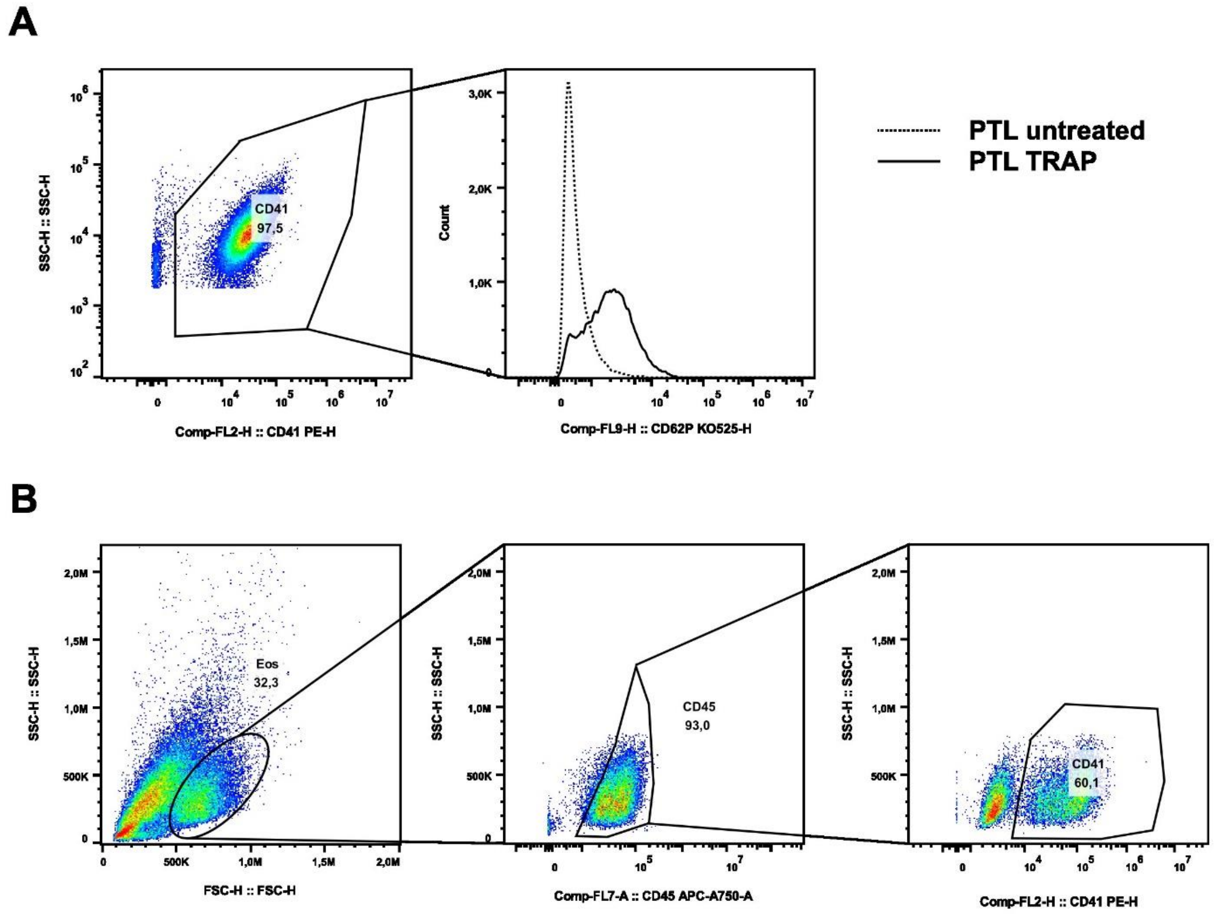

Supplementary figure 4: Gating strategy of aggregate formation assay with PLTs. (A) Aggregate formation was measured via flow cytometry. Eosinophilic cells were identified by size and granularity (FSC/SSC) and CD45-staining. PLT-bound eosinophils were determined as $CD41^+$ eosinophilic cells. **(B)** Platelet activation was verified by flow cytometry. PLTs were identified by CD41-staining. Activation was confirmed by a MFI-shift of CD62P.

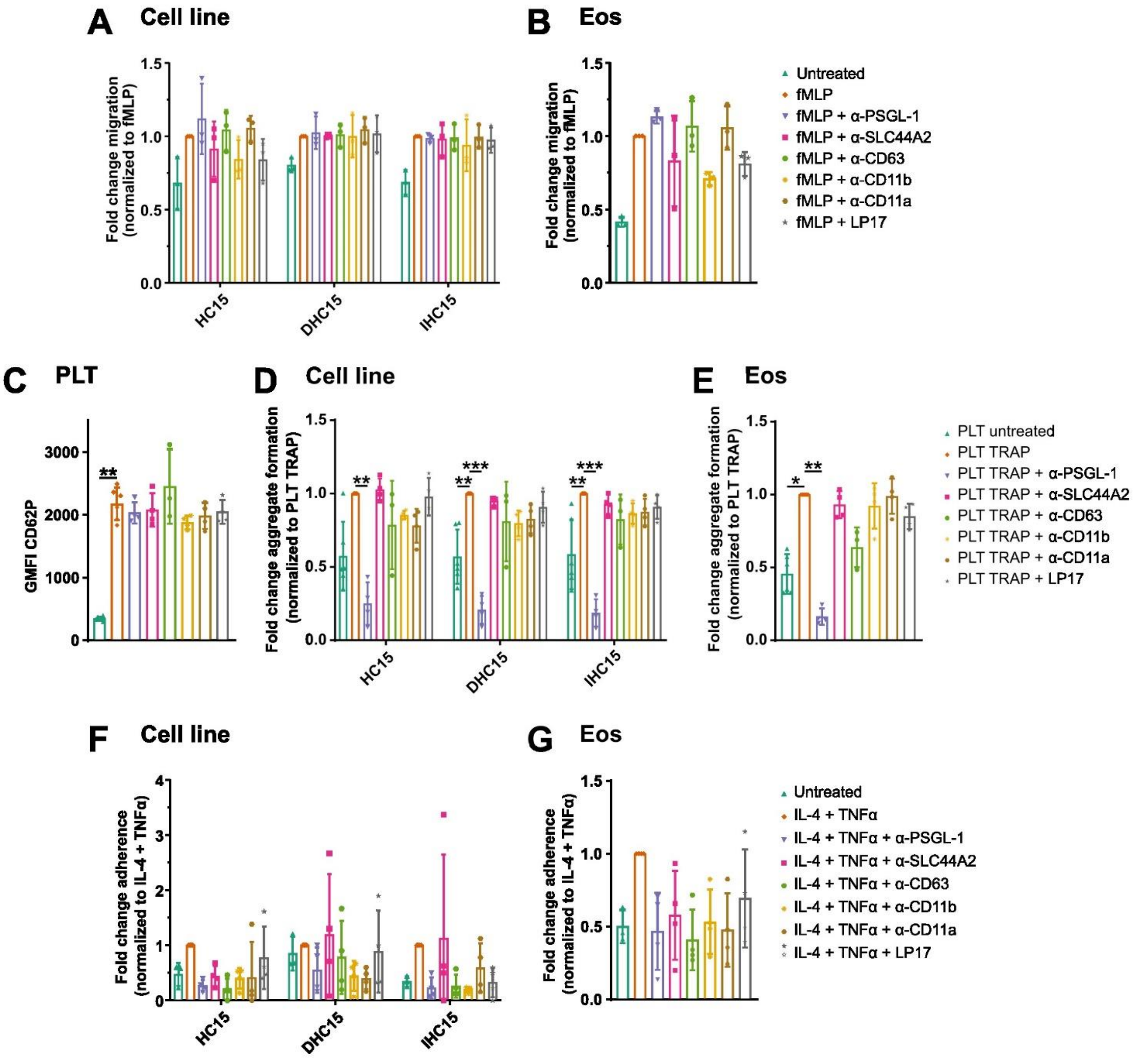

Supplementary figure 5: PLT-eosinophil aggregate formation is significantly dependent on PSGL-1 expression on eosinophilic cells. In each experiment, cell line cells or Eos were preincubated with specific blocking reagents for 10 min (blocking antibodies) or 2 h (LP17), respectively. **(A-B)** Transwell cell migration assay. After stimulation with 100 nM fMLP for 60 min, cells in the lower chamber were counted (N=3). **(A)** Cell line migration. **(B)** Eos migration. **(C-E)** Aggregate formation assay with PLTs. Resting or activated PLTs were incubated with cells in a 200:1 ratio and aggregate formation was analyzed by flow cytometry (N=3-6). **(C)** PLT activation was confirmed by measurement of CD62P expression. **(D)** PLT-cell line aggregate formation. **(E)** PLT-Eos aggregate formation. **(F-G)** Cell adherence to HUVECs in a PLT-rich environment. CFSE-stained cell line cells or Eos were incubated with resting or activated HUVECs with PLTs for 30 min, fixed, DAPI-stained and identified by immunofluorescent microscopy.**(F)** Cell line adherence to HUVECs. **(G)** Eos adherence to HUVECs. Data is depicted as bar graphs. * shows significance vs. treated. *P<0.05, **P<0.01, ***P<0.001; Kruskal-Wallis test with Dunn's multiple comparisons.

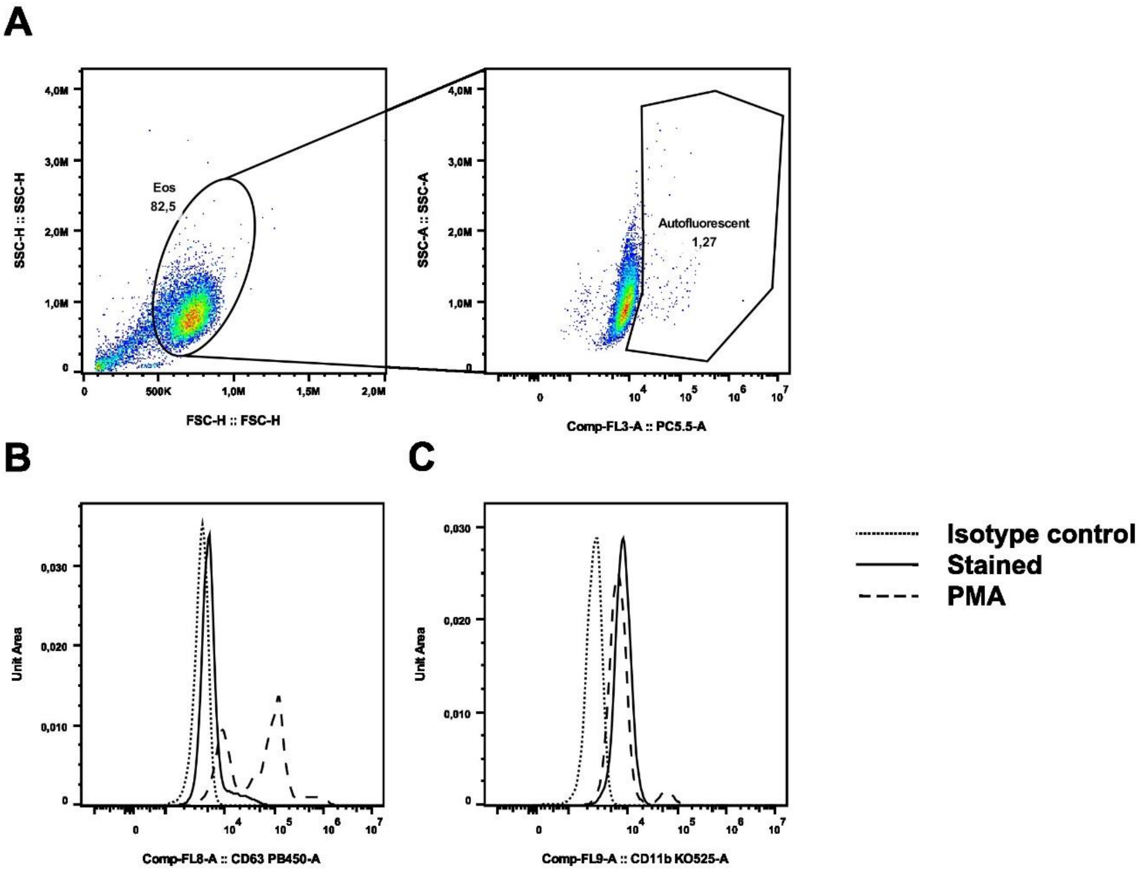

Supplementary figure 6: Gating strategy for determintation of CD63 and CD11b levels on eosinophilic cells activated with PMA. (A) Eosinophilic cells were identified by size and granularity. Autofluorescent cells were gated out. **(B-C)** The surface marker levels of CD63 **(C)** and CD11b **(D)** were determined by a MFI-shift of the respective marker.

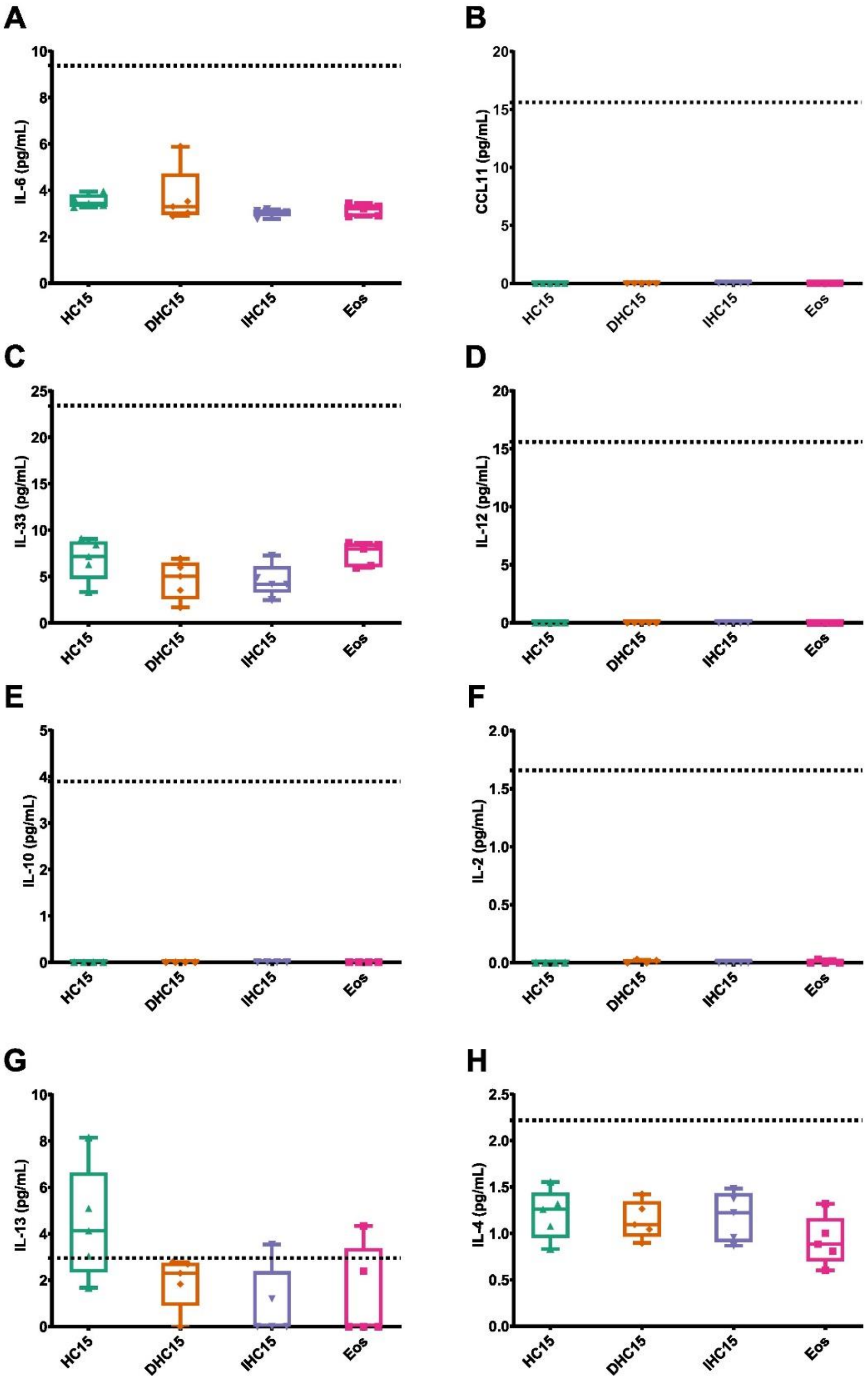

Supplementary figure 7: Inflammatory milieu of untreated eosinophilic cells. Cytokine secretion levels of eosinophilic cells (N=5) were measured either by ELISA (IL-6 **(A)**, CCL11 **(B)**, IL-33 **(C)**, IL-12 **(D)**, IL10 **(E)**) or by Legendplex assay (IL-2 **(F)**, IL-13 **(G)**, IL-4 **(H)**). The visualized detection limit is the lowest value of the standard curve provided by the manufacturer. Data are depicted as boxplots.
