# Supplementary material for: The HL-60 clone 15 cell line as a model for leukocyte migration–possibilities and limitations
Source: Front Immunol. 2025 May 27;16:1515993. doi: 10.3389/fimmu.2025.1515993 (PMC12148902; doi:10.3389/fimmu.2025.1515993)
Supplement: Supplementary file 2 [file DataSheet2.pdf]

Supplementary material

Supplementary tables

Supplementary table 1: List of gene IDs from proteins exclusively shared between IHC15 cells and Eos.

Supplementary table 2: List of gene IDs from proteins exclusively shared between DHC15 cells and Eos.

Supplementary table 3: List of gene IDs from proteins exclusively abundant in Eos.

Supplementary table 4: Statistics details of Figure 3.

Supplementary table 5: Statistics details of Figure 4.

Supplementary table 6: Statistics details of Figure 5.

Supplementary table 7: Statistics details of Figure 6.

Supplementary table 8: Statistics details of Figure 7.

Supplementary table 9: Statistics details of Supplementary figure 5.

Supplementary table 10: Statistics details of Figure 8.

Supplementary table 11: Statistics details of Figure 9.

Supplementary table 12: Loading table of the top 15 proteins contributing to component 1 of the PCA analysis.

Supplementary table 13: Loading table of the top 15 proteins contributing to component 2 of the PCA analysis.

Supplementary table 1: List of gene IDs from proteins exclusively shared between IHC15 cells and Eos.

SYNM	CYTIP	HPGDS	CPNE3
RTN2	ENDOD1	CA1	APOD
HCK;LYN;SRC;YES1	CD48	HSPA5;HSPA8	SRC
CCL5	TPSAB1;TPSB2	PTX3	MARK2;MARK3
GSTM2;GSTM4	CCR3	S100A10	BCL2L1
KLC1;KLC2;KLC4	TLE5	MMRN1	RAB33A
RFTN1	SEPTIN2;SEPTIN7	SLC27A3	EEPD1
TOR1AIP2	SEPTIN1;SEPTIN5	NRGN	LPP
PLD4	BORCS6	HSH2D	SFXN1;SFXN3
ADGRE2;ADGRE3	TRIM4	ARNT2	PARP4
PLCL2	SIGLEC7	RABGAP1	RIPOR2
RAB19	IGLL5	DDX58	FN1
IGKV4-1	BPGM	PF4V1	ORM2
MAOB	GSTM2	IDUA	GMPR
CLTCL1	NEXN	AKAP13	PGM2L1
DHRS11	CAMK1D	FGGY	DHRS9
HTATIP2	PAG1	DAPK2	PPM1H
PTGDR2	VNN1	IGLV1-51	CD74
SPARC	PYGM	ACADS	GP5
RBKS	RAB27B	PLXNC1	TNFAIP8
NUDT14	VWF	H1-0	RHOC
HMOX1	ACYP2	CAPN2	MMP8
PXN	GNG10	SULT1A1	PPP2CA;PPP4C
GSTM4	WIPI1	ZDHHC20	DPPA3
TANK	ACSF2	LHPP	DLGAP4

Supplementary table 2: List of gene IDs from proteins exclusively shared between DHC15 cells and Eos.

RTN2	VWF	OSCAR	LPP
APOD	BPGM	PLD4	ACSF2
SRC	TPSAB1;TPSB2	HSH2D	TRIM4
PTX3	GNG10	ARNT2	PAG1
S100A10	TLE5	DAPK2	PARP4
SEPTIN2;SEPTIN7	NHSL2	RABGAP1	PTGDR2
ZDHHC20	TOR1AIP2	IGKV3-15;IGKV3-7;IGKV3D-7	PDLIM1
PHOSPHO1	SEPTIN1;SEPTIN5	DDX58	CA1
BORCS6	DHRS9	SERPINA3	HMOX1
SFXN1;SFXN3	ADGRE2;ADGRE3	MX2	MARK2;MARK3
PLCL2	SIGLEC7	SULT1A1	AKAP13
DLGAP4	HPGDS	DHRS11	ARID3B
CPNE3	FN1	NPEPL1	NRGN
CD48	HSPA5;HSPA8	TANK	FGGY
PYGM	CCL5	LGALS12	LHPP
ORM2	GSTM2;GSTM4	TNFAIP8	VNN1
GP5	CCR3	TTR	HCK;LYN;SRC;YES1
BCL2L1	KLC1;KLC2;KLC4	ACYP2	PPP2CA;PPP4C
CHIT1	RFTN1	CTTN	WIPI1
PGM2L1	EEPD1	LY6G6F	LILRA6
ESAM			

Supplementary table 3: List of gene IDs from proteins exclusively abundant in different cell types.

Gene IDs from proteins exclusively abundant in HC15

**ENPP3**	SKP2	OLR1

Gene IDs from proteins exclusively abundant in DHC15

**LILRA5**	STOML3

Gene IDs from proteins exclusively abundant in IHC15

**LRRK2**	TXNRD3	LILRA3	DPYSL4

Gene IDs from proteins exclusively abundant in Eos

IGKV2D-30	IGHG2;IGHG4	GSTM1	ADPRH	C2orf88
IGKV3-11;IGKV3D-11	IGHG3	CSF1	GNG11	INPP5K
IGKV3D-20	IGHG4	SULT1A3;SULT1A4	HBG2	IFT27
IGKV3-20;IGKV3D-20	IGHA1	CLU	ADAM8	CYRIA
IGHV4-30-4;IGHV4-34;IGHV4-38-2;IGHV4-39;IGHV4-59;IGHV4-61	HLA-A;HLA-H	SPTB	LCN2	CPVL
FAAH	HLA-DRA	FRK;SRC	AKR1C1	FN3K
MAPK13	HLA-DRB1	GP1BB	CALD1	DPEP2
TGFB1I1	HBD	ACP5	LGALS3BP	RGCC
KALRN	APOA1	SELL	DMTN	GP6
PFKFB2	SLC4A1	GP9	SCRN1	SMPD3
PGLYRP1	APCS	IDO1	ITIH4	TUBA8
DDAH1	APOH	CD36	CD226	SH3BGRL2
DDAH1;DDAH2	RBP4	ITGA2	LGALSL	PITPNC1
CAVIN2	AMBP	ALOX12	C9orf64	DAPP1
MPIG6B	PF4	GNAZ	CYB5R2	CHKB
CFB	PF4;PF4V1	HLA-DPA1	ACOT1	NCKAP1
KNG1	HPX	CA4	TREML1	EMILIN1
IGKV1-5	HRG	MYL9	LILRB2	IGHV1-3;IGHV1-46;IGHV1-69;IGHV1-69D;IGHV1-8

Gene IDs from proteins exclusively abundant in Eos

IGHV3-7	A1BG	PDE4A;PDE4D	APPL2	TDRD15
IGHG1;IGHG2;IGHG3	SERPING1	GALNS	SYNE1;SYNE2	AGT
IGHG1;IGHG2;IGHG3;IGHG4	SERPIND1	SNCA	CD200R1	IGKV3-20
IGHG1;IGHG3;IGHG4	APOA4	ACAA2	SSH3	IGLV1-47
IGHG2	PROS1	HAAO	TMEM40	SNTB2
IGHG2;IGHG3	TPM1;TPM2	SERPINB9	CMTM5	IRAG1
IGHG2;IGHG3;IGHG4	CFH	RAB13	SORT1	IGLC7
HLA-F	PON1	C4B	IGHG1;IGHG2	IGLV3-21;IGLV3-9
AFM				

Supplementary table 4: Statistics details of Figure 3.

		Significance testing					Effect size					Multiple comparisons						
		**n**	**DF**	**p**	**p.signif**	**method**	**effsize**	**conf.low**	**conf.high**	**method**	**magnitude**	**group1**	**group2**	**n1**	**n2**	**p.adj**	**p.adj.signif**	**method**
IL5R	**all cells**	22	3	0.0011	**	Kruskal-Wallis	0.7290	0.4000	0.8900	eta2	large	Eos	HC15	7	5	0.0012	**	Dunn with bonferroni correction
												Eos	DHC15	7	5	0.6371	ns	Dunn with bonferroni correction
												Eos	IHC15	7	5	0.2203	ns	Dunn with bonferroni correction
	**only cell line**	15	2	0.0082	#	Kruskal-Wallis	0.6350	0.2700	0.8600	eta2	large	HC15	DHC15	5	5	0.0112	#	Dunn with bonferroni correction
												DHC15	IHC15	5	5	1.0000	ns	Dunn with bonferroni correction
												HC15	IHC15	5	5	0.0486	#	Dunn with bonferroni correction
EMR1	**all cells**	22	3	0.0018	**	Kruskal-Wallis	0.6652	0.2800	0.9200	eta2	large	Eos	HC15	7	5	0.0008	***	Dunn with bonferroni correction
												Eos	DHC15	7	5	1.0000	ns	Dunn with bonferroni correction
												Eos	IHC15	7	5	0.3557	ns	Dunn with bonferroni correction
	**only cell line**	15	2	0.0068	##	Kruskal-Wallis	0.6650	0.3000	0.8800	eta2	large	HC15	DHC15	5	5	0.0071	##	Dunn with bonferroni correction
												DHC15	IHC15	5	5	1.0000	ns	Dunn with bonferroni correction
												HC15	IHC15	5	5	0.0710	ns	Dunn with bonferroni correction
Siglec8	**all cells**	22	3	0.0046	**	Kruskal-Wallis	0.5566	0.2300	0.8200	eta2	large	Eos	HC15	7	5	0.3044	ns	Dunn with bonferroni correction
												Eos	DHC15	7	5	0.0159	*	Dunn with bonferroni correction
												Eos	IHC15	7	5	0.0134	*	Dunn with bonferroni correction
	**only cell line**	15	2	0.2810	ns	Kruskal-Wallis	0.0450	-0.1500	0.6500	eta2	small	HC15	DHC15	5	5	0.5373	ns	Dunn with bonferroni correction
												DHC15	IHC15	5	5	1.0000	ns	Dunn with bonferroni correction
												HC15	IHC15	5	5	0.4719	ns	Dunn with bonferroni correction
**CCR3**	**all cells**	33	3	0.0072	**	Kruskal-Wallis	0.2387	-0.0046	0.5600	eta2	large	Eos	HC15	9	8	0.0112	*	Dunn with bonferroni correction
												Eos	DHC15	9	8	0.0291	*	Dunn with bonferroni correction
												Eos	IHC15	9	8	0.2294	ns	Dunn with bonferroni correction
	**only cell line**	24	2	0.4050	ns	Kruskal-Wallis	-0.0065	-0.0600	0.2800	eta2	small	HC15	DHC15	8	8	1.0000	ns	Dunn with bonferroni correction
												DHC15	IHC15	8	8	1.0000	ns	Dunn with bonferroni correction
												HC15	IHC15	8	8	0.5722	ns	Dunn with bonferroni correction

Supplementary table 5: Statistics details of Figure 4.

		Significance testing					Effect size					Multiple comparisons						
		**n**	**DF**	**p**	**p.signif**	**method**	**effsize**	**conf.low**	**conf.high**	**method**	**magnitude**	**group1**	**group2**	**n1**	**n2**	**p.adj**	**p.adj.signif**	**method**
SPI1	**all cells**	21	3	0.2420	ns	Kruskal-Wallis	0.0659	-0.1000	0.6100	eta2	moderate	Eos	HC15	6	5	1.0000	ns	Dunn with bonferroni correction
												Eos	DHC15	6	5	1.0000	ns	Dunn with bonferroni correction
												Eos	IHC15	6	5	0.7232	ns	Dunn with bonferroni correction
	**only cell line**	15	2	0.3100	ns	Kruskal-Wallis	0.0283	-0.1500	0.7000	eta2	small	HC15	DHC15	5	5	0.8665	ns	Dunn with bonferroni correction
												DHC15	IHC15	5	5	1.0000	ns	Dunn with bonferroni correction
												HC15	IHC15	5	5	0.4127	ns	Dunn with bonferroni correction
GATA1	**all cells**	22	3	0.0017	**	Kruskal-Wallis	0.6724	0.3500	0.9200	eta2	large	Eos	HC15	7	5	0.0012	**	Dunn with bonferroni correction
												Eos	DHC15	7	5	0.1143	ns	Dunn with bonferroni correction
												Eos	IHC15	7	5	1.0000	ns	Dunn with bonferroni correction
	**only cell line**	15	2	0.0063	##	Kruskal-Wallis	0.6783	0.3200	0.8800	eta2	large	HC15	DHC15	5	5	0.2691	ns	Dunn with bonferroni correction
												DHC15	IHC15	5	5	0.4127	ns	Dunn with bonferroni correction
												HC15	IHC15	5	5	0.0044	##	Dunn with bonferroni correction
ID2	**all cells**	20	3	0.0095	**	Kruskal-Wallis	0.4693	0.1600	0.7600	eta2	large	Eos	HC15	6	4	0.0601	ns	Dunn with bonferroni correction
												Eos	DHC15	6	5	0.0333	*	Dunn with bonferroni correction
												Eos	IHC15	6	5	0.0467	*	Dunn with bonferroni correction
	**only cell line**	14	2	0.9970	ns	Kruskal-Wallis	-0.1662	-0.1600	0.5900	eta2	large	HC15	DHC15	4	5	1.0000	ns	Dunn with bonferroni correction
												DHC15	IHC15	5	5	1.0000	ns	Dunn with bonferroni correction
												HC15	IHC15	4	5	1.0000	ns	Dunn with bonferroni correction

Supplementary table 6: Statistics details of Figure 5.

		Significance testing					Effect size					Multiple comparisons						
		**n**	**DF**	**p**	**p.signif**	**method**	**effsize**	**conf.low**	**conf.high**	**method**	**magnitude**	**group1**	**group2**	**n1**	**n2**	**p.adj**	**p.adj.signif**	**method**
PRG2	**all cells**	22	3	0.0023	**	Kruskal-Wallis	0.6404	0.3600	0.8300	eta2	large	Eos	HC15	6	5	0.3121	ns	Dunn with bonferroni correction
												Eos	DHC15	6	5	0.0029	**	Dunn with bonferroni correction
												Eos	IHC15	6	5	0.0188	*	Dunn with bonferroni correction
	**only cell line**	15	2	0.1140	ns	Kruskal-Wallis	0.1950	-0.1000	0.7200	eta2	large	HC15	DHC15	5	5	0.1209	ns	Dunn with bonferroni correction
												DHC15	IHC15	5	5	1.0000	ns	Dunn with bonferroni correction
												HC15	IHC15	5	5	0.5373	ns	Dunn with bonferroni correction
EPX	**all cells**	22	3	0.0006	***	Kruskal-Wallis	0.7928	0.6000	0.9000	eta2	large	Eos	HC15	6	5	0.8594	ns	Dunn with bonferroni correction
												Eos	DHC15	6	5	0.0064	**	Dunn with bonferroni correction
												Eos	IHC15	6	5	0.0016	**	Dunn with bonferroni correction
	**only cell line**	15	2	0.0082	##	Kruskal-Wallis	0.6350	0.2500	0.8500	eta2	large	HC15	DHC15	5	5	0.0486	#	Dunn with bonferroni correction
												DHC15	IHC15	5	5	1.0000	ns	Dunn with bonferroni correction
												HC15	IHC15	5	5	0.0112	#	Dunn with bonferroni correction
**EMBP**	**all cells**	24	3	0.7000	ns	Kruskal-Wallis	-0.0788	-0.1300	0.4000	eta2	moderate	Eos	HC15	5	6	1.0000	ns	Dunn with bonferroni correction
												Eos	DHC15	5	6	1.0000	ns	Dunn with bonferroni correction
												Eos	IHC15	5	6	1.0000	ns	Dunn with bonferroni correction
	**only cell line**	18	2	0.5230	ns	Kruskal-Wallis	-0.0468	-0.1300	0.4800	eta2	small	HC15	DHC15	6	6	1.0000	ns	Dunn with bonferroni correction
												DHC15	IHC15	6	6	1.0000	ns	Dunn with bonferroni correction
												HC15	IHC15	6	6	0.7684	ns	Dunn with bonferroni correction
**EPX**	**all cells**	24	3	0.0060	**	Kruskal-Wallis	0.4727	0.1300	0.8100	eta2	large	Eos	HC15	5	6	0.0035	**	Dunn with bonferroni correction
												Eos	DHC15	5	6	0.0729	ns	Dunn with bonferroni correction
												Eos	IHC15	5	6	0.1413	ns	Dunn with bonferroni correction
	**only cell line**	18	2	0.2290	ns	Kruskal-Wallis	0.0632	-0.1200	0.6700	eta2	moderate	HC15	DHC15	6	6	0.5831	ns	Dunn with bonferroni correction
												DHC15	IHC15	6	6	1.0000	ns	Dunn with bonferroni correction
												HC15	IHC15	6	6	0.3143	ns	Dunn with bonferroni correction

Supplementary table 7: Statistics details of Figure 6.

		Significance testing					Effect size					Multiple comparisons						
		**n**	**DF**	**p**	**p.signif**	**method**	**effsize**	**conf.low**	**conf.high**	**method**	**magnitude**	**group1**	**group2**	**n1**	**n2**	**p.adj**	**p.adj.signif**	**method**
SELPLG	**all cells**	22	3	0.0061	**	Kruskal-Wallis	0.5221	0.2500	0.7700	eta2	large	Eos	HC15	6	5	0.0223	*	Dunn with bonferroni correction
												Eos	DHC15	6	5	0.0223	*	Dunn with bonferroni correction
												Eos	IHC15	6	5	0.0590	ns	Dunn with bonferroni correction
	**only cell line**	15	2	0.8870	ns	Kruskal-Wallis	-0.1467	-0.1600	0.5700	eta2	large	HC15	DHC15	5	5	1.0000	ns	Dunn with bonferroni correction
												DHC15	IHC15	5	5	1.0000	ns	Dunn with bonferroni correction
												HC15	IHC15	5	5	1.0000	ns	Dunn with bonferroni correction
CD40	**all cells**	22	3	0.1120	ns	Kruskal-Wallis	0.1665	-0.1000	0.6800	eta2	large	Eos	HC15	7	5	0.1538	ns	Dunn with bonferroni correction
												Eos	DHC15	7	5	0.4721	ns	Dunn with bonferroni correction
												Eos	IHC15	7	5	0.7298	ns	Dunn with bonferroni correction
	**only cell line**	15	2	0.8270	ns	Kruskal-Wallis	-0.1350	-0.1600	0.4500	eta2	moderate	HC15	DHC15	5	5	1.0000	ns	Dunn with bonferroni correction
												DHC15	IHC15	5	5	1.0000	ns	Dunn with bonferroni correction
												HC15	IHC15	5	5	1.0000	ns	Dunn with bonferroni correction
CD63	**all cells**	22	3	0.0093	**	Kruskal-Wallis	0.4722	0.1700	0.7900	eta2	large	Eos	HC15	7	5	0.0297	*	Dunn with bonferroni correction
												Eos	DHC15	7	5	0.0349	*	Dunn with bonferroni correction
												Eos	IHC15	7	5	0.1341	ns	Dunn with bonferroni correction
	**only cell line**	15	2	0.7560	ns	Kruskal-Wallis	-0.1200	-0.1600	0.5800	eta2	moderate	HC15	DHC15	5	5	1.0000	ns	Dunn with bonferroni correction
												DHC15	IHC15	5	5	1.0000	ns	Dunn with bonferroni correction
												HC15	IHC15	5	5	1.0000	ns	Dunn with bonferroni correction
ITGAM	**all cells**	22	3	0.0051	**	Kruskal-Wallis	0.5452	0.2600	0.8000	eta2	large	Eos	HC15	6	5	0.0077	**	Dunn with bonferroni correction
												Eos	DHC15	6	5	0.0431	*	Dunn with bonferroni correction
												Eos	IHC15	6	5	0.0799	ns	Dunn with bonferroni correction
	**only cell line**	15	2	0.5950	ns	Kruskal-Wallis	-0.0800	-0.1600	0.5300	eta2	moderate	HC15	DHC15	5	5	1.0000	ns	Dunn with bonferroni correction
												DHC15	IHC15	5	5	1.0000	ns	Dunn with bonferroni correction
												HC15	IHC15	5	5	0.9666	ns	Dunn with bonferroni correction

		Significance testing					Effect size					Multiple comparisons						
		**n**	**DF**	**p**	**p.signif**	**method**	**effsize**	**conf.low**	**conf.high**	**method**	**magnitude**	**group1**	**group2**	**n1**	**n2**	**p.adj**	**p.adj.signif**	**method**
SLC44A2	**all cells**	22	3	0.0009	***	Kruskal-Wallis	0.7532	0.5400	0.8800	eta2	large	Eos	HC15	6	5	0.0003	***	Dunn with bonferroni correction
												Eos	DHC15	6	5	0.1206	ns	Dunn with bonferroni correction
												Eos	IHC15	6	5	0.3899	ns	Dunn with bonferroni correction
	**only cell line**	15	2	0.0113	#	Kruskal-Wallis	0.5800	0.1700	0.8100	eta2	large	HC15	DHC15	5	5	0.0710	ns	Dunn with bonferroni correction
												DHC15	IHC15	5	5	1.0000	ns	Dunn with bonferroni correction
												HC15	IHC15	5	5	0.0140	#	Dunn with bonferroni correction
ITGAL	**all cells**	22	3	0.1210	ns	Kruskal-Wallis	0.1567	-0.0600	0.7500	eta2	large	Eos	HC15	7	5	1.0000	ns	Dunn with bonferroni correction
												Eos	DHC15	7	5	0.1033	ns	Dunn with bonferroni correction
												Eos	IHC15	7	5	1.0000	ns	Dunn with bonferroni correction
	**only cell line**	15	2	0.0652	ns	Kruskal-Wallis	0.2883	-0.0100	0.7700	eta2	large	HC15	DHC15	5	5	0.1687	ns	Dunn with bonferroni correction
												DHC15	IHC15	5	5	0.1017	ns	Dunn with bonferroni correction
												HC15	IHC15	5	5	1.0000	ns	Dunn with bonferroni correction
TREM1	**all cells**	22	3	0.0146	*	Kruskal-Wallis	0.4179	0.1300	0.7000	eta2	large	Eos	HC15	5	5	0.3363	ns	Dunn with bonferroni correction
												Eos	DHC15	5	5	0.0354	*	Dunn with bonferroni correction
												Eos	IHC15	5	5	0.0299	*	Dunn with bonferroni correction
	**only cell line**	15	2	0.4300	ns	Kruskal-Wallis	-0.0262	-0.1600	0.5700	eta2	small	HC15	DHC15	5	5	0.9773	ns	Dunn with bonferroni correction
												DHC15	IHC15	5	4	1.0000	ns	Dunn with bonferroni correction
												HC15	IHC15	5	4	0.6770	ns	Dunn with bonferroni correction

Supplementary Table 8: Statistics details of Figure 7.

		Significance testing						Effect size					Multiple comparisons						
		effect	DFn	DFd	p	p.signif	method	eff.size	conf.low	conf.high	method	magnitude	group1	group2	n1	n2	p.adj	p.adj. signif	method
**Migration**	**Cell line**	Diff	2	24	0.0000	####	Two-way ANOVA	0.5740	0.2993	0.6870	partial eta2	large	HC15 UT	DHC15 UT	5	5	0.0627	ns	Tukey HSD with bonferroni correction
		Treatment	1	24	0.0250	*	Two-way ANOVA	0.1920	0.0135	0.3927	partial eta2	moderate	DHC15 UT	IHC15 UT	5	5	1.0000	ns	Tukey HSD with bonferroni correction
		Diff: Treatment	2	24	0.3800	ns	Two-way ANOVA	0.0780	0.0000	0.2288	partial eta2	moderate	HC15 UT	IHC15 UT	5	5	0.0398	*	Tukey HSD with bonferroni correction
													HC15 T	DHC15 T	5	5	0.1630	ns	Tukey HSD with bonferroni correction
													DHC15 T	IHC15 T	5	5	0.3010	ns	Tukey HSD with bonferroni correction
													HC15 T	IHC15 T	5	5	0.0013	**	Tukey HSD with bonferroni correction
													HC15 UT	HC15 T	5	5	0.8940	ns	Tukey HSD with bonferroni correction
													DHC15 UT	DHC15 T	5	5	0.9920	ns	Tukey HSD with bonferroni correction
													IHC15 UT	IHC15 T	5	5	0.1630	ns	Tukey HSD with bonferroni correction
	**Eos**							1.0000	0.7431	1.0000	Cliff's delta	very large	UT	T	4	5	0.0179	*	Mann-Whitney U with bonferroni correction
**Aggregate formation**	**Cell line**	Diff	2	40	0.7720	ns	Two-way ANOVA	0.0130	0.0000	0.0746	partial eta2	small	HC15 UT	DHC15 UT	7	7	0.9620	ns	Tukey HSD with bonferroni correction
		Treatment	1	40	0.0000	****	Two-way ANOVA	0.3950	0.1948	0.5365	partial eta2	large	DHC15 UT	IHC15 UT	7	8	1.0000	ns	Tukey HSD with bonferroni correction
		Diff: Treatment	2	40	0.2310	ns	Two-way ANOVA	0.0710	0.0000	0.1919	partial eta2	moderate	HC15 UT	IHC15 UT	7	8	0.9830	ns	Tukey HSD with bonferroni correction
													HC15 T	DHC15 T	8	8	0.5610	ns	Tukey HSD with bonferroni correction
													DHC15 T	IHC15 T	8	8	0.9320	ns	Tukey HSD with bonferroni correction
													HC15 T	IHC15 T	8	8	0.9780	ns	Tukey HSD with bonferroni correction
													HC15 UT	HC15 T	7	8	0.5830	ns	Tukey HSD with bonferroni correction
													DHC15 UT	DHC15 T	7	8	0.0026	**	Tukey HSD with bonferroni correction
													IHC15 UT	IHC15 T	8	8	0.0369	*	Tukey HSD with bonferroni correction
	**Eos**							1.0000	0.9286	1.0000	Cliff's delta	very large	UT	T	6	8	0.0024	**	Mann-Whitney U with bonferroni correction
	**PTL**							1.0000	0.9522	1.0000	Cliff's delta	very large	UT	T	6	8	0.0002	***	Mann-Whitney U with bonferroni correction

		Significance testing						Effect size					Multiple comparisons						
		effect	DFn	DFd	p	p.signif	method	eff.size	conf.low	conf.high	method	magnitude	group1	group2	n1	n2	p.adj	p.adj. signif	method
**Adherence**	**Cell line**	Diff	2	69	0.8630	ns	Two-way ANOVA	0.0040	0.0000	0.0292	partial eta2	small	HC15 UT	DHC15 UT	13	13	1.0000	ns	Tukey HSD with bonferroni correction
		Treatment	1	69	0.0530	ns	Two-way ANOVA	0.0530	0.0000	0.1565	partial eta2	small	DHC15 UT	IHC15 UT	13	13	0.9910	ns	Tukey HSD with bonferroni correction
		Diff: Treatment	2	69	0.5650	ns	Two-way ANOVA	0.0160	0.0000	0.0734	partial eta2	small	HC15 UT	IHC15 UT	13	13	0.9970	ns	Tukey HSD with bonferroni correction
													HC15 T	DHC15 T	13	13	0.0530	ns	Tukey HSD with bonferroni correction
													DHC15 T	IHC15 T	13	13	0.9420	Ns	Tukey HSD with bonferroni correction
													HC15 T	IHC15 T	13	13	1.0000	ns	Tukey HSD with bonferroni correction
													HC15 UT	HC15 T	13	13	0.9310	ns	Tukey HSD with bonferroni correction
													DHC15 UT	DHC15 T	13	13	0.3680	ns	Tukey HSD with bonferroni correction
													IHC15 UT	IHC15 T	13	13	0.9970	ns	Tukey HSD with bonferroni correction
	**Eos**							0.9167	0.5198	0.9881	Cliffs Delta	very large	UT	T	6	6	0.0098	**	Mann-Whitney U with bonferroni correction

Supplementary table 9: Statistics details of Supplementary figure 5.

		Significance testing					Effect size					Multiple comparisons						
		n	DF	p	p.signif	method	effsize	conf.low	conf.high	method	magnitude	group1	group2	n1	n2	p.adj	p.adj.signif	method
**Migration**	**HC15**	24	7	0,0564	ns	Kruskal-Wallis	0,4198	0,2200	0,8600	eta2	large	UT	T	3	3	0,7869	ns	Dunn with bonferroni correction
												T	PSGL-1	3	3	1,0000	ns	Dunn with bonferroni correction
												T	SLC44A2	3	3	1,0000	ns	Dunn with bonferroni correction
												T	CD63	3	3	1,0000	ns	Dunn with bonferroni correction
												T	CD11b	3	3	1,0000	ns	Dunn with bonferroni correction
												T	CD11a	3	3	1,0000	ns	Dunn with bonferroni correction
												T	LP17	3	3	1,0000	ns	Dunn with bonferroni correction
	**DHC15**	24	7	0,3220	ns	Kruskal-Wallis	0,0700	-0,1100	0,7800	eta2	moderate	UT	T	3	3	1,0000	ns	Dunn with bonferroni correction
												T	PSGL-1	3	3	1,0000	ns	Dunn with bonferroni correction
												T	SLC44A2	3	3	1,0000	ns	Dunn with bonferroni correction
												T	CD63	3	3	1,0000	ns	Dunn with bonferroni correction
												T	CD11b	3	3	1,0000	ns	Dunn with bonferroni correction
												T	CD11a	3	3	1,0000	ns	Dunn with bonferroni correction
												T	LP17	3	3	1,0000	ns	Dunn with bonferroni correction
	**IHC15**	24	7	0,267	ns	Kruskal-Wallis	0,1126	-0,0900	0,8300	eta2	moderate	UT	T	3	3	0,2608	ns	Dunn with bonferroni correction
												T	PSGL-1	3	3	1,0000	ns	Dunn with bonferroni correction
												T	SLC44A2	3	3	1,0000	ns	Dunn with bonferroni correction
												T	CD63	3	3	1,0000	ns	Dunn with bonferroni correction
												T	CD11b	3	3	1,0000	ns	Dunn with bonferroni correction
												T	CD11a	3	3	1,0000	ns	Dunn with bonferroni correction
												T	LP17	3	3	1,0000	ns	Dunn with bonferroni correction
	**Eos**	24	7	0,0161	*	Kruskal-Wallis	0,6385	0,4000	0,9500	eta2	large	UT	T	3	3	0,6782	ns	Dunn with bonferroni correction
												T	PSGL-1	3	3	1,0000	ns	Dunn with bonferroni correction
												T	SLC44A2	3	3	1,0000	ns	Dunn with bonferroni correction
												T	CD63	3	3	1,0000	ns	Dunn with bonferroni correction
												T	CD11b	3	3	1,0000	ns	Dunn with bonferroni correction
												T	CD11a	3	3	1,0000	ns	Dunn with bonferroni correction
												T	LP17	3	3	1,0000	ns	Dunn with bonferroni correction

		Significance testing					Effect size					Multiple comparisons							
		n	DF	p	p.signif	method	effsize	conf.low	conf.high	method	magnitude	group1	group2	n1	n2	p.adj	p.adj.signif	method
**Aggregate formation**	**PLT**	35	7	0,0087	**	Kruskal-Wallis	0,4382	0,2300	0,7800	eta2	large	UT	T	6	6	0,0097	**	Dunn with bonferroni correction
												T	PSGL-1	6	4	1,0000	ns	Dunn with bonferroni correction
												T	SLC44A2	6	4	1,0000	ns	Dunn with bonferroni correction
												T	CD63	6	3	1,0000	ns	Dunn with bonferroni correction
												T	CD11b	6	4	1,0000	ns	Dunn with bonferroni correction
												T	CD11a	6	4	1,0000	ns	Dunn with bonferroni correction
												T	LP17	6	4	1,0000	ns	Dunn with bonferroni correction
	**HC15**	35	7	0,0014	**	Kruskal-Wallis	0,6085	0,3800	0,9000	eta2	large	UT	T	6	6	0,2044	ns	Dunn with bonferroni correction
												T	PSGL-1	6	4	0,0049	**	Dunn with bonferroni correction
												T	SLC44A2	6	4	1,0000	ns	Dunn with bonferroni correction
												T	CD63	6	3	1,0000	ns	Dunn with bonferroni correction
												T	CD11b	6	4	1,0000	ns	Dunn with bonferroni correction
												T	CD11a	6	4	1,0000	ns	Dunn with bonferroni correction
												T	LP17	6	4	1,0000	ns	Dunn with bonferroni correction
	**DHC15**	35	7	0,0004	***	Kruskal-Wallis	0,7348	0,5600	0,9400	eta2	large	UT	T	6	6	0,0060	**	Dunn with bonferroni correction
												T	PSGL-1	6	4	0,0003	***	Dunn with bonferroni correction
												T	SLC44A2	6	4	1,0000	ns	Dunn with bonferroni correction
												T	CD63	6	3	1,0000	ns	Dunn with bonferroni correction
												T	CD11b	6	4	0,2795	ns	Dunn with bonferroni correction
												T	CD11a	6	4	0,6441	ns	Dunn with bonferroni correction
												T	LP17	6	4	1,0000	ns	Dunn with bonferroni correction
	**IHC15**	35	7	0,0009	***	Kruskal-Wallis	0,6510	0,4400	0,9100	eta2	large	UT	T	6	6	0,0094	**	Dunn with bonferroni correction
												T	PSGL-1	6	4	0,0003	***	Dunn with bonferroni correction
												T	SLC44A2	6	4	1,0000	ns	Dunn with bonferroni correction
												T	CD63	6	3	1,0000	ns	Dunn with bonferroni correction
												T	CD11b	6	4	0,8623	ns	Dunn with bonferroni correction
												T	CD11a	6	4	0,9479	ns	Dunn with bonferroni correction
												T	LP17	6	4	1,0000	ns	Dunn with bonferroni correction

		Significance testing					Effect size					Multiple comparisons						
		**n**	**DF**	**p**	**p.signif**	**method**	**effsize**	**conf.low**	**conf.high**	**method**	**magnitude**	**group1**	**group2**	**n1**	**n2**	**p.adj**	**p.adj.signif**	**method**
**Aggregate formation**	**Eos**	35	7	0,0003	***	Kruskal-Wallis	0,7567	0,5900	0,9400	eta2	large	UT	T	6	6	0,0267	*	Dunn with bonferroni correction
												T	PSGL-1	6	4	0,0042	**	Dunn with bonferroni correction
												T	SLC44A2	6	4	1,0000	ns	Dunn with bonferroni correction
												T	CD63	6	3	0,7086	ns	Dunn with bonferroni correction
												T	CD11b	6	4	1,0000	ns	Dunn with bonferroni correction
												T	CD11a	6	4	1,0000	ns	Dunn with bonferroni correction
												T	LP17	6	4	1,0000	ns	Dunn with bonferroni correction
**Adherence**	**HC15**	31	7	0,0437	*	Kruskal-Wallis	0,324	0,13	0,81	eta2	large	UT	T	4	3	1,0000	ns	Dunn with bonferroni correction
												T	PSGL-1	4	4	0,2114	ns	Dunn with bonferroni correction
												T	SLC44A2	4	4	1,0000	ns	Dunn with bonferroni correction
												T	CD63	4	4	0,0798	ns	Dunn with bonferroni correction
												T	CD11b	4	4	1,0000	ns	Dunn with bonferroni correction
												T	CD11a	4	4	0,2114	ns	Dunn with bonferroni correction
												T	LP17	4	4	1,0000	ns	Dunn with bonferroni correction
	**DHC15**	31	7	0,309	ns	Kruskal-Wallis	0,0556	-0,0400	0,6600	eta2	small	UT	T	4	3	1,0000	ns	Dunn with bonferroni correction
												T	PSGL-1	4	4	1,0000	ns	Dunn with bonferroni correction
												T	SLC44A2	4	4	1,0000	ns	Dunn with bonferroni correction
												T	CD63	4	4	1,0000	ns	Dunn with bonferroni correction
												T	CD11b	4	4	1,0000	ns	Dunn with bonferroni correction
												T	CD11a	4	4	0,7302	ns	Dunn with bonferroni correction
												T	LP17	4	4	1,0000	ns	Dunn with bonferroni correction
	**IHC15**	31	7	0,0851	ns	Kruskal-Wallis	0,2394	0,0500	0,7500	eta2	large	UT	T	4	3	1,0000	ns	Dunn with bonferroni correction
												T	PSGL-1	4	4	0,1483	ns	Dunn with bonferroni correction
												T	SLC44A2	4	4	1,0000	ns	Dunn with bonferroni correction
												T	CD63	4	4	0,3328	ns	Dunn with bonferroni correction
												T	CD11b	4	4	0,1881	ns	Dunn with bonferroni correction
												T	CD11a	4	4	1,0000	ns	Dunn with bonferroni correction
												T	LP17	4	4	0,9857	ns	Dunn with bonferroni correction

		Significance testing					Effect size					Multiple comparisons						
		**n**	**DF**	**p**	**p.signif**	**method**	**effsize**	**conf.low**	**conf.high**	**method**	**magnitude**	**group1**	**group2**	**n1**	**n2**	**p.adj**	**p.adj.signif**	**method**
**Adherence**	**Eos**	31	7	0,11	ns	Kruskal-Wallis	0,2048	0,02	0,72	eta2	large	UT	T	4	3	0,9095	ns	Dunn with bonferroni correction
												T	PSGL-1	4	4	0,3562	ns	Dunn with bonferroni correction
												T	SLC44A2	4	4	1,0000	ns	Dunn with bonferroni correction
												T	CD63	4	4	0,0981	ns	Dunn with bonferroni correction
												T	CD11b	4	4	1,0000	ns	Dunn with bonferroni correction
												T	CD11a	4	4	0,3018	ns	Dunn with bonferroni correction
												T	LP17	4	4	1,0000	ns	Dunn with bonferroni correction

Supplementary table 10: Statistics details of Figure 8.

		**Significance testing**						**Effect size**					**Multiple comparisons**						
		**effect**	**DFn**	**DFd**	**p**	**p.signif**	**method**	**eff.size**	**conf.low**	**conf.high**	**method**	**magnitude**	**group1**	**group2**	**n1**	**n2**	**p.adj**	**p.adj. signif**	**method**
**CCL5**	**Cell line**	Diff	2	24	0.0000	####	Two-way ANOVA	0.8030	0.6389	0.8557	partial eta2	large	HC15 UT	DHC15 UT	5	5	0.9690	ns	Tukey HSD with bonferroni correction
		Treatment	1	24	0.0020	**	Two-way ANOVA	0.3390	0.0930	0.5208	partial eta2	large	DHC15 UT	IHC15 UT	5	5	0.0674	ns	Tukey HSD with bonferroni correction
		Diff: Treatment	2	24	0.0009	***	Two-way ANOVA	0.4410	0.1515	0.5841	partial eta2	large	HC15 UT	IHC15 UT	5	5	0.0119	#	Tukey HSD with bonferroni correction
													HC15 T	DHC15 T	5	5	0.7390	ns	Tukey HSD with bonferroni correction
													DHC15 T	IHC15 T	5	5	0.0000	####	Tukey HSD with bonferroni correction
													HC15 T	IHC15 T	5	5	0.0000	####	Tukey HSD with bonferroni correction
													HC15 UT	HC15 T	5	5	1.0000	ns	Tukey HSD with bonferroni correction
													DHC15 UT	DHC15 T	5	5	0.9930	ns	Tukey HSD with bonferroni correction
													IHC15 UT	IHC15 T	5	5	0.0001	***	Tukey HSD with bonferroni correction
	**Eos**							0.7600	-0.0882	0.9693	Cliff's delta	large	UT	T	8	8	0.0519	ns	Mann-Whitney U with bonferroni correction
**EPX**	**Cell line**	Diff	2	24	0.2220	ns	Two-way ANOVA	0.1180	0.0000	0.2829	partial eta2	moderate	HC15 UT	DHC15 UT	5	5	0.9420	ns	Tukey HSD with bonferroni correction
		Treatment	1	24	0.3660	ns	Two-way ANOVA	0.0340	0.0000	0.2024	partial eta2	small	DHC15 UT	IHC15 UT	5	5	0.2250	ns	Tukey HSD with bonferroni correction
		Diff: Treatment	2	24	0.1990	ns	Two-way ANOVA	0.1260	0.0000	0.2926	partial eta2	moderate	HC15 UT	IHC15 UT	5	5	0.7170	ns	Tukey HSD with bonferroni correction
													HC15 T	DHC15 T	5	5	0.9060	ns	Tukey HSD with bonferroni correction
													DHC15 T	IHC15 T	5	5	1.0000	ns	Tukey HSD with bonferroni correction
													HC15 T	IHC15 T	5	5	0.9580	ns	Tukey HSD with bonferroni correction
													HC15 UT	HC15 T	5	5	0.9200	ns	Tukey HSD with bonferroni correction
													DHC15 UT	DHC15 T	5	5	0.9300	ns	Tukey HSD with bonferroni correction
													IHC15 UT	IHC15 T	5	5	0.6280	ns	Tukey HSD with bonferroni correction
	**Eos**							0.4400	-0.3656	0.8687	Cliff's delta	medium	UT	T	5	5	0.3100	ns	Mann-Whitney U with bonferroni correction

		Significance testing						Effect size					Multiple comparisons						
		**effect**	**DFn**	**DFd**	**p**	**p.signif**	**method**	**eff.size**	**conf.low**	**conf.high**	**method**	**magnitude**	**group1**	**group2**	**n1**	**n2**	**p.adj**	**p.adj. signif**	**method**
**CD63**	**Cell line**	Diff	2	57	0.0360	#	Two-way ANOVA	0.1100	0.0039	0.2260	partial eta2	moderate	HC15 UT	DHC15 UT	11	11	1.0000	ns	Tukey HSD with bonferroni correction
		Treatment	1	57	0.0060	**	Two-way ANOVA	0.1230	0.0206	0.2571	partial eta2	moderate	DHC15 UT	IHC15 UT	11	11	0.8920	ns	Tukey HSD with bonferroni correction
		Diff: Treatment	2	57	0.4700	ns	Two-way ANOVA	0.0260	0.0000	0.1009	partial eta2	small	HC15 UT	IHC15 UT	11	11	0.8560	ns	Tukey HSD with bonferroni correction
													HC15 T	DHC15 T	11	11	0.8980	ns	Tukey HSD with bonferroni correction
													DHC15 T	IHC15 T	11	8	0.1090	ns	Tukey HSD with bonferroni correction
													HC15 T	IHC15 T	11	8	0.5660	ns	Tukey HSD with bonferroni correction
													HC15 UT	HC15 T	11	11	0.6840	ns	Tukey HSD with bonferroni correction
													DHC15 UT	DHC15 T	11	11	0.1120	ns	Tukey HSD with bonferroni correction
													IHC15 UT	IHC15 T	11	8	0.9740	ns	Tukey HSD with bonferroni correction
	**Eos**							1.0000	0.8178	1.0000	Cliff's delta	large	UT	T	9	9	0.0005	***	Mann-Whitney U with bonferroni correction
**CD11b**	**Cell line**	Diff	2	59	0.0050	##	Two-way ANOVA	0.1640	0.0322	0.2872	partial eta2	large	HC15 UT	DHC15 UT	11	11	0.9980	ns	Tukey HSD with bonferroni correction
		Treatment	1	59	0.0002	****	Two-way ANOVA	0.2100	0.0686	0.3405	partial eta2	large	DHC15 UT	IHC15 UT	11	11	0.9870	ns	Tukey HSD with bonferroni correction
		Diff: Treatment	2	59	0.0620	ns	Two-way ANOVA	0.0900	0.0000	0.1993	partial eta2	moderate	HC15 UT	IHC15 UT	11	11	0.8870	ns	Tukey HSD with bonferroni correction
													HC15 T	DHC15 T	10	11	0.0051	##	Tukey HSD with bonferroni correction
													DHC15 T	IHC15 T	11	11	0.9930	ns	Tukey HSD with bonferroni correction
													HC15 T	IHC15 T	10	11	0.0245	#	Tukey HSD with bonferroni correction
													HC15 UT	HC15 T	11	10	0.9990	ns	Tukey HSD with bonferroni correction
													DHC15 UT	DHC15 T	11	11	0.0043	**	Tukey HSD with bonferroni correction
													IHC15 UT	IHC15 T	11	11	0.1100	ns	Tukey HSD with bonferroni correction
	**Eos**							-0.1200	-0.7555	0.6319	Cliff's delta	negligible	UT	T	9	9	0.3400	ns	Mann-Whitney U with bonferroni correction

Supplementary table 11: Statistics details of Figure 9.

		Significance testing					Effect size					Multiple comparisons						
		**n**	**DF**	**p**	**p.signif**	**method**	**effsize**	**conf.low**	**conf.high**	**method**	**magnitude**	**group1**	**group2**	**n1**	**n2**	**p.adj**	**p.adj.signif**	**method**
**CCL5**	**all cells**	23	3	0.0002	***	Kruskal-Wallis	0.8899	0.7200	0.9300	eta2	large	Eos	HC15	8	5	0.7811	ns	Dunn with bonferroni correction
												Eos	DHC15	8	5	0.0217	*	Dunn with bonferroni correction
												Eos	IHC15	8	5	0.0002	***	Dunn with bonferroni correction
	**only cell line**	15	2	0.0019	##	Kruskal-Wallis	0.8750	0.7200	0.8900	eta2	large	HC15	DHC15	5	5	0.2313	ns	Dunn with bonferroni correction
												DHC15	IHC15	5	5	0.2313	ns	Dunn with bonferroni correction
												HC15	IHC15	5	5	0.0012	##	Dunn with bonferroni correction
**EPX**	**all cells**	23	3	0.0042	**	Kruskal-Wallis	0.5365	0.2500	0.7900	eta2	large	Eos	HC15	5	5	0.0277	*	Dunn with bonferroni correction
												Eos	DHC15	5	5	0.0046	**	Dunn with bonferroni correction
												Eos	IHC15	5	5	0.4150	ns	Dunn with bonferroni correction
	**only cell line**	15	2	0.1140	ns	Kruskal-Wallis	0.1950	-0.1200	0.7400	eta2	large	HC15	DHC15	5	5	1.0000	ns	Dunn with bonferroni correction
												DHC15	IHC15	5	5	0.1209	ns	Dunn with bonferroni correction
												HC15	IHC15	5	5	0.5373	ns	Dunn with bonferroni correction
**IL-8**	**all cells**	23	3	0.0010	***	Kruskal-Wallis	0.7010	0.4700	0.8900	eta2	large	Eos	HC15	5	5	0.6369	ns	Dunn with bonferroni correction
												Eos	DHC15	5	5	0.0682	ns	Dunn with bonferroni correction
												Eos	IHC15	5	5	0.0005	***	Dunn with bonferroni correction
	**only cell line**	15	2	0.0092	##	Kruskal-Wallis	0.6150	0.2500	0.8500	eta2	large	HC15	DHC15	5	5	0.6880	ns	Dunn with bonferroni correction
												DHC15	IHC15	5	5	0.1980	ns	Dunn with bonferroni correction
												HC15	IHC15	5	5	0.0071	##	Dunn with bonferroni correction

Supplementary table 12: Loading table of the top 15 proteins contributing to component 1 of the PCA analysis.

#	Contribution to component	Protein group	First protein name	First protein function (extracted from Uniprot)
**1**	0.1272	TRFL	Lactotransferrin	Iron binding transport protein.
**2**	0.1166	ECP	Eosinophil cationic protein	Mediates tyrosine nitration of secondary granule proteins in mature resting eosinophils.
**3**	0.0940	DEF3	Neutrophil defensin 3	Effector molecule of the innate immune system.
**4**	0.0907	CEAM1;CEAM8	Carcinoembryonic antigen-related cell adhesion molecule 1	Cell adhesion protein that mediates homophilic cell adhesion.
**5**	0.0857	HPT	Haptoglobin	Captures free plasma hemoglobin for hepatic recycling.
**6**	0.0843	CAP7	Azurocidin	Neutrophil granule-derived antibacterial and chemotactic glycoprotein.
**7**	0.0796	ANXA3	Annexin A3	Inhibitor of phospholipase A2.
**8**	0.0786	CDD	Cytidine deaminase	Scavenges exogenous and endogenous cytidine.
**9**	0.0781	ITAM	Integrin alpha-M	Adhesion molecule, part of Integrin αMβ2.
**10**	0.0780	ITAM;ITAX	Integrin alpha-M	Adhesion molecule, part of Integrin αMβ2.
**11**	0.0687	ELNE	Neutrophil elastase	Serine protease that modifies the functions of natural killer cells, monocytes and granulocytes.
**12**	0.0665	CATG	Cathepsin G	Serine protease with trypsin- and chymotrypsin-like specificity.
**13**	0.0645	PADI2	Protein-arginine deiminase type-2	Catalyzes the deimination of arginine residues of proteins.
**14**	0.0595	ALBU	Albumin	Major zinc transporter in plasma.
**15**	0.0587	HS71A;HS71B;HS71L	Heat shock 70 kDa protein 1A	Molecular chaperone implicated in a wide variety of cellular processes.

Supplementary table 13: Loading table of the top 15 proteins contributing to component 2 of the PCA analysis.

#	Contribution to component	Protein group	First protein name	First protein function (extracted from Uniprot)
**1**	0.2096	PTTG	Pituitary tumor-transforming gene 1 protein-interacting protein	May facilitate PTTG1 nuclear translocation.
**2**	0.1688	RASA2	Ras GTPase-activating protein 2	Inhibitory regulator of the Ras-cyclic AMP pathway.
**3**	0.1398	ISOC1	Isochorismatase domain-containing protein 1	No known function
**4**	0.1257	SYNE1	Nesprin-1	Link between organelles and the actin cytoskeleton.
**5**	0.1104	NMNA1	Nicotinamide/nicotinic acid mononucleotide adenylyltransferase 1	Catalyzes the formation of NAD^+ from nicotinamide mononucleotide.
**6**	0.1044	RAB10;RAB13;RAB15;RAB1A;RAB1B;RAB1C;RAB8A;RAB8B	Ras-related protein Rab-10	Key regulator of intracellular membrane trafficking.
**7**	0.0937	PI42A	Phosphatidylinositol 5-phosphate 4-kinase type-2 alpha	Catalyzes the phosphorylation of phosphatidylinositol 5-phosphate.
**8**	0.0848	ARL1	ADP-ribosylation factor-like protein 1	GTP-binding protein that recruits several effectors tot he trans-Golgi network.
**9**	0.0834	NRBF2	Nuclear receptor-binding factor 2	May modulate transcriptional activation.
**10**	0.0808	HBB;HBD	Hemoglobin subunit beta	Involved in oxygen transport from the lung to the various peripheral tissues.
**11**	0.0806	CAH2	Carbonic anhydrase 2	Catalyzes the reversible hydration of carbon dioxide.
**12**	0.0773	PP4P1	Type 1 phosphatidylinositol 4,5-bisphosphate 4-phosphatase	Catalyzes the hydrolysis of phosphatidylinositol-4,5-bisphosphate.
**13**	0.0770	DHX57	Putative ATP-dependent RNA helicase DHX57	Probable ATP-binding RNA helicase.
**14**	0.0767	TRI56	E3 ubiquitin-protein ligase TRIM56	E3 ubiquitin-protein ligase that plays a key role in innate antiviral immunity.
**15**	0.0732	MYO1E	Unconventional myosin-Ie	Actin-based motor molecule with ATPase activity.
